# Overexpression of *VIRMA* confers vulnerability to breast cancers via the m^6^A-dependent regulation of unfolded protein response

**DOI:** 10.1007/s00018-023-04799-4

**Published:** 2023-05-19

**Authors:** Quintin Lee, Renhua Song, Dang Anh Vu Phan, Natalia Pinello, Jessica Tieng, Anni Su, James M. Halstead, Alex C. H. Wong, Michelle van Geldermalsen, Bob S.-L. Lee, Bowen Rong, Kristina M. Cook, Mark Larance, Renjing Liu, Fei Lan, Jessamy C. Tiffen, Justin J.-L. Wong

**Affiliations:** 1 Epigenetics and RNA Biology Program Centenary Institute, The University of Sydney, Camperdown, NSW 2006 Australia; 2 Faculty of Medicine and Health, The University of Sydney, Camperdown, NSW 2006 Australia; 3 Gene and Stem Cell Therapy Program Centenary Institute, The University of Sydney, Camperdown, NSW 2006 Australia; 4 Victor Chang Cardiac Research Institute, Sydney, NSW 2010 Australia; 5 Shanghai Key Laboratory of Medical Epigenetics, International Laboratory of Medical Epigenetics and Metabolism, Ministry of Science and Technology, Institutes of Biomedical Sciences, Fudan University, Shanghai, China; 6 Key Laboratory of Carcinogenesis and Cancer Invasion, Ministry of Education, Liver Cancer Institute, Zhongshan Hospital, Fudan University, Shanghai, 200032 China; 7 Charles Perkins Centre, University of Sydney, Camperdown, NSW 2006 Australia; 8 School of Clinical Medicine, Faculty of Medicine and Health, UNSW Sydney, Kensington, NSW 2052 Australia; 9 Melanoma Epigenetics Laboratory Centenary Institute, The University of Sydney, Camperdown, NSW 2006 Australia; 10 Locked Bag 6, Newtown, NSW 2042 Australia

**Keywords:** N^6^-methyladenosine (m^6^a), Messenger RNA, VIRMA, Unfolded protein response, Endoplasmic reticulum stress, Breast cancer

## Abstract

Virilizer-like m^6^A methyltransferase-associated protein (VIRMA) maintains the stability of the m^6^A writer complex. Although VIRMA is critical for RNA m^6^A deposition, the impact of aberrant VIRMA expression in human diseases remains unclear. We show that VIRMA is amplified and overexpressed in 15–20% of breast cancers. Of the two known VIRMA isoforms, the nuclear-enriched full-length but not the cytoplasmic-localised N-terminal VIRMA promotes m^6^A-dependent breast tumourigenesis in vitro and in vivo. Mechanistically, we reveal that VIRMA overexpression upregulates the m^6^A-modified long non-coding RNA, *NEAT1*, which contributes to breast cancer cell growth. We also show that VIRMA overexpression enriches m^6^A on transcripts that regulate the unfolded protein response (UPR) pathway but does not promote their translation to activate the UPR under optimal growth conditions. Under stressful conditions that are often present in tumour microenvironments, VIRMA-overexpressing cells display enhanced UPR and increased susceptibility to death. Our study identifies oncogenic VIRMA overexpression as a vulnerability that may be exploited for cancer therapy.

**Supplementary Information:**

The online version contains supplementary material available at 10.1007/s00018-023-04799-4.

## Introduction

Modifications to RNA molecules regulate normal cell development and are implicated in cancers. Of over 170 RNA modifications reported to date [1], methylation of N^6^-adenosine (m^6^A) residues is the most prevalent internal modification on messenger RNAs (mRNAs) and is present on non-coding RNAs [2, 3]. m^6^A is deposited by a core m^6^A methyltransferase complex comprising Methyltransferase-3 (METTL3), Methyltransferase-14 (METTL14) and Wilm's Tumor Associated Protein (WTAP) [4]. While mutations of m^6^A regulators are rare (0.02–8.07%) [5–7], aberrant expressions of these proteins confer oncogenic potential in diverse cancers [8–10].

Other auxiliary proteins are also important in regulating optimal m^6^A deposition on RNAs [4, 11]. However, the impact of genetic alteration and aberrant expression of genes encoding these proteins in cancer development, maintenance and response to therapy is poorly understood. One of the least studied auxiliary m^6^A-depositing proteins is the Virilizer-like methyltransferase, VIRMA. VIRMA has been reported as the scaffold that brings together all components of the m^6^A methyltransferase complex for selective m^6^A methylation in the 3′ untranslated region (3′ UTR) and near stop codons [12]. Depletion of VIRMA resulted in a greater global loss of m^6^A mRNA methylation than the individual depletion of METTL3, METTL14 or WTAP [11, 12]. Thus, aberrantly expressed VIRMA may alter m^6^A methylation of many more transcripts than aberrantly expressed core m^6^A methyltransferases to cause human diseases, including cancer.

Recent studies have reported VIRMA as an oncogenic factor [13–21]. Most of them proposed the oncogenic potential of VIRMA from loss of function studies, whereby VIRMA depletion resulted in attenuation of cancer growth and/or metastasis. This conclusion needs to be interpreted with caution because VIRMA is an essential gene for cell survival [22, 23]; therefore, the loss of viability in either healthy or malignant cells, is expected with the loss of VIRMA.

VIRMA exists in two distinct isoforms: the full-length isoform comprising 1812 amino acids (aa) and the N-terminal isoform that contains only the first 1130 aa of the full-length isoform [12]. While studies have shown that VIRMA overexpression promotes growth and/or metastasis in cancers [18–21], it was not clear which isoforms of VIRMA are ectopically overexpressed. Understanding the involvement of distinct VIRMA isoforms in tumourigenesis is important in determining whether there is one predominant isoform that contributes to cancer development. A recent study identified an m^6^A-independent role of VIRMA in breast tumourigenesis by overexpressing the shorter (N-terminal) isoform of VIRMA but not the more highly-expressed full-length isoform [17]. Whether overexpressing the full-length VIRMA affects breast cancer pathology warrants an investigation. It is tantalising to speculate that the full-length VIRMA isoform will exert an m^6^A-dependent role in cancer development and maintenance. The influence of VIRMA overexpression and downstream consequences of m^6^A methylation changes on tumour microenvironment and chemotherapeutic response in cancer remains to be determined.

By determining the prevalence of *VIRMA* gene alterations and investigating the role of distinct VIRMA isoforms in breast cancer, we identify a unique m^6^A-dependent oncogenic role of full-length VIRMA under optimal cell growth conditions. However, in breast cancer cells overexpressing full-length VIRMA, hypoxia and endoplasmic reticulum (ER) stress enhance an m^6^A-dependent unfolded protein response (UPR) and decrease cell viability. Our findings indicate that VIRMA overexpression in breast cancer may be an “Achilles Heel” that could be exploited for precision treatment of cancer cells with chemotherapeutic agents that trigger UPR and ER stress response.

## Materials and methods

### Patient data

The clinicopathological, mutation, copy number variation, gene expression and survival data from the TCGA (Firehose Legacy) and METABRIC breast cancer cohorts were obtained via cBioportal [24, 25]. PAM50 status was assigned to samples as previously described [26]. The Kaplan–Meier analysis and the log-rank test were used to determine overall survival (OS) via the SPSS statistical package, version 24.0 (IBM, Corp.).

### Cell lines and culture conditions

*VIRMA* mRNA expression in breast cancer cell lines were extracted from the Cancer Cell Line Encyclopedia (Broad Institute) [27] to determine cell lines with low, intermediate and high *VIRMA* expression for subsequent experiments. SKBR3, MDA-MB-231, MDA-MB-453, MCF7, AU565, HS578T, and HEK293T cells were obtained from the American Tissue Culture Collection (ATCC). SKBR3, MDA-MB-231, and MDA-MB-453 cells were maintained in RPMI media (ThermoFisher cat# 22400089) supplemented with 10% fetal bovine serum (GE Life Sciences cat# SH30084.03). AU565 and HS578T cells were cultured in DMEM media and supplements mentioned above with the addition of 1 mM sodium pyruvate (Gibco cat# 11360-070) and 10 µg/mL of insulin (Novo nordisk cat#1331415), respectively. MCF7 cells were cultured in α-MEM supplemented with 10% (v/v) fetal bovine serum (FBS), 7.5% (v/v) sodium bicarbonate and 1 × (v/v) Glutamax. HEK293T cells were maintained in DMEM (ThermoFisher cat# 12430054) supplemented with 10% fetal bovine serum (GE Life Sciences cat# SH30084.03). All media contained 0.1 mg/mL penicillin/streptomycin (ThermoFisher cat#15140148) and all cell lines were maintained at 37 °C in the presence of 5% CO_2_.

### Construction of VIRMA-overexpressing cell lines

HA-tagged full-length VIRMA (VIRMA FL) and N-terminal (N-term) VIRMA with eGFP separated by p2A were synthesised with the inclusion of specific restriction sites, and subcloned into the FUW lentiviral vector at Gene Universal (Delaware, USA). Sequences and cloning strategy are detailed in the Supplementary Methods. The control vector (eGFP Con) contained the enhanced green fluorescence protein (eGFP) coding sequence downstream of the human ubiquitin C (UbC) promoter in FUW. These individual engineered FUW vectors were packaged into lentivirus via co-transfection with plasmids encoding VSV-G, Gag/Pol and Rev into HEK293T cells. After incubation for 48 h, the supernatant was collected, centrifuged and passed through 0.45 µm filter to remove cell debris. For viral transduction, cells were seeded at 4 × 10^5^ cells per well in a 6-well plate and were allowed to adhere overnight before new media containing 4 μg/mL polybrene and 500 μL of unconcentrated virus was added. The plates were centrifuged at 500×*g* for 90 min at room temperature. The virus-containing media was then replaced with fresh media. After 48 h in culture, eGFP-expressing cells were purified using fluorescence-activated cell sorting and expanded for subsequent experiments.

### Lentiviral shRNAs transduction

shRNAs targeting VIRMA were designed, synthesised and subcloned into the pLKO.1-puro vector (Addgene). shRNA targeting sequences are listed in Table S1. Lentiviruses were generated using HEK293T cells and supernatant containing virus was collected and filtered with 0.45 µm filter. Viral transduction was performed as described for the generation of VIRMA-overexpressing cell lines. Post-infection, virus-containing media were replaced with fresh media containing 2 μg/mL puromycin. Puromycin selection was performed for up to 10 days until no viable cell remained in the non-transduced control plate. Transduced cells that survived were pooled and expanded for downstream assays.

### Cell proliferation assays

For cell doubling calculation, cells were seeded in 6-well plates at 1 × 10^4^ cells per well and maintained at 37 °C with 5% CO_2_. After 2, 4, 6, and 8 days, cells were harvested and counted using a cell hemocytometer. For 3-(4,5-dimethylthiazol-2-yL)-2,5-Diphenyltetrazolium Bromide MTT assays, cells were seeded at 1–2 × 10^3^ cells per well in a 96-well plate. The MTT solution was added at a final concentration of 0.5 mg/mL. For slow growing cells, SKBR3 and AU565, the absorbance was measured at 570 nm using the Tecan Infinite M1000 plate reader at day 0 (24 h post-seeding), 2, 4, 6 and 8 days. For fast growing HS578T cells, absorbance was measured at day 0 (24 h post-seeding), 1, 2, 3 and 4. Proliferation of the MDA-MB-231 cells was assessed using the Cell Counting Kit 8 (CCK8) kit (Abcam cat# ab228554) according to the manufacturer’s instructions. CCK8 solution (10 µL) was added to each well of a 96-well plate seeded with 1 × 10^3^ cells per 100 µL of media. Absorbance at 450 nm was measured each day for five consecutive days beginning from day 0 (24 h post-seeding).

### Clonogenicity assay

For AU565 and SKBR3, 3000 cells were seeded in a 10 cm culture plate and maintained in culture for 14 days. For HS578T, MDA-MB-231 and MCF7, cells were seeded at 1000 cells per plate and cultured for 7 days. Colonies were fixed with ice-cold methanol and stained with Giemsa (Sigma Aldrich cat# GS500). Images were taken using the ChemiDoc™ Imaging System (Bio-Rad). Colonies were counted using Image J.

### Western blot

Cells were lysed in 1 × RIPA buffer with protease inhibitors. Protein concentration was measured with Pierce™ BCA Protein Assay Kit (ThermoFisher, cat#23225) following manufacturer’s instructions. Protein lysates were loaded onto precast SDS-PAGE gels (Invitrogen) and subjected to electrophoresis before being transferred onto polyvinylidene difluoride (PVDF; Millipore, cat# IPVH00010) membranes. Membranes were blocked with 5% (w/v) skim milk or 5% (w/v) bovine serum albumin for 1 h at room temperature and incubated overnight with primary antibodies: VIRMA (1:1000; Cell Signaling Technology cat#88358), METTL3 (1:1000; Abcam cat#ab195352), METTL14 (1:1000; Sigma cat#HPA038002), WTAP (1:1000; Proteintech cat#10200-1-AP), p-EIF2α (Ser51) (1:1000; Cell Signaling Technology cat#3398), EIF2α (1:1000; Cell Signaling Technology cat#3179), ATF4 (1:1000; Cell Signaling Technology cat#11815), α-Tubulin (1:5000; Santa Cruz cat#sc-5286), LDHA (1:2500; Cell Signaling Technology cat#2012), LMNB1 (1;5000, Abcam cat#16048), GAPDH (1:5000; Abcam cat#ab8245), HA.11 Epitope Tag (1:2500; BioLegend cat#901502). Following washes in 1 × PBS, membranes were incubated with HRP-conjugated secondary anti-rabbit or anti-mouse antibody (1:5000; Chemicon AP182P or AP192P) and exposed using SuperSignal™ West Pico PLUS Chemiluminescent Substrate (Pierce, cat#34578) on a ChemiDoc™ Imaging System (Bio-Rad). Relative expression of protein normalised to loading control was quantified using the ImageJ software (National Institutes of Health).

### Mouse xenografts

Eight-week old NSG (NOD.Cg-Prkdc-scid.Il2rg-tm1Wjl) female mice were ordered from the Australian Resources Centre (Western Australia). Mice were acclimatised at the Centenary Institute Animal Facility for a minimum of 7 days after initial arrival. Prior to tumour injection, mice were anaesthetised by ketamine/xylazine by intraperitoneal injection. Subsequently, the fur surrounding the 4th mammary fat pad on the right was removed using the hair removal cream. MDA-MB-231 cells (5 × 10^6^) in 100 µL of 1:1 HBSS:Matrigel were then injected subcutaneously into the 4th right mammary fat pad using 27 g insulin needles. Mice were injected intraperitoneally with the reversal atipamezole to improve the recovery from anaesthesia. Mice were monitored twice weekly by assessing body condition, measuring body weights and tumour sizes. The frequency of monitoring was increased to daily when tumours reached > 500 mm^3^ in size. Mice were killed when tumours reached > 1000 mm^3^ in size and the relevant organs were harvested for analysis. All experiments have been approved by the Sydney Local Health District Animal Welfare Committee (Protocol # 2019/032).

### Immunohistochemistry

Tissues were fixed in 4% paraformaldehyde overnight, embedded in paraffin and sectioned at 5 μm. Sections were deparaffinised and rehydrated and stained for H&E based on standard protocol. For immunohistochemistry staining, sections were deparaffinised and stained with primary antibodies against CD31 (1:500, Abcam, USA) for endothelial cells, and Ki67 for cell proliferation (1:100, Invitrogen, USA) followed by HRP conjugated secondary antibodies (1:500, Invitrogen, USA). Samples were incubated with Metal Enhanced DAB Substrate Kit (ThermoFisher, cat#34065) and counterstained with Harris’ Haematoxylin solution. Slides were dehydrated and mounted with DPX mounting media.

### Nuclear and cytoplasmic fractionation

Nuclear and cytoplasmic fractionation was performed with 2 × 10^6^ cells using the NE-PER™ Nuclear and Cytoplasmic Extraction Reagents Kit (ThermoFisher, cat# 78833) according to the manufacturer’s instructions.

### RNA m^6^A quantification by LC–MS/MS

Quantification of global RNA m^6^A levels by LC–MS/MS was performed as previously described. Briefly, TRIzol (Invitrogen, cat# 15596018) was used to isolate total RNA from eGFP control, VIRMA FL and VIRMA N-term samples. Polyadenylated RNAs were then enriched by oligo d(T)25 magnetic beads (New England BioLabs, cat# S1550S), followed by removal of rRNA with RiboMinus Eukaryote Kit (Invitrogen, cat# A15026). mRNA was measured using the Qubit™ RNA HS Assay kit (ThermoFisher, cat# Q32852) and digested by nuclease P1 (Sigma, cat# N8630) in 20 µL buffer containing 25 mM NaCl (Sigma, cat# S5150-1L), 2.5 mM ZnCl2 at 37 °C for 2 h. Following the addition of NH_4_HCO_3_ for a final concentration of 0.1 M (Sigma, cat# A6141-500G) and alkaline phosphatase (Sigma, cat# A5931-200UN), the solution was incubated at 37 °C for an additional 2 h. The solution was centrifuged at 10,000*g* for 10 min at 4 °C, and 10 µL of the solution was injected into LC–MS/MS. Quantification was performed with reference to the standard curve obtained from pure nucleoside standards, and the ratio of m^6^A to A was then determined.

### m^6^A-RIP-sequencing

m^6^A-RIP-sequencing was performed according to the published “Refined RIP-seq” protocol with several modifications[28]. Total RNA (5 μg) was fragmented into ~ 200 nucleotide-long fragments via incubation with ZnCl2 at 70 °C for 3 min. Fragmented RNA was precipitated, and the RNA size distribution was assessed using RNA 6000 Nano Bioanalyzer kit (Agilent cat# 5067-1511). After setting aside 500 ng of fragmented RNA as input control, the rest of the RNA was subjected to two rounds of immunoprecipitation, each for 2 h, using anti-m6A antibody (Merck, cat#ABE572) conjugated to protein-A and –G magnetic beads (ThermoFisher, cat# 10002D and 10004D). Extensive washing was performed on immunoprecipitated RNA and elution from beads was carried out using RLT buffer and RNeasy mini kit (Qiagen, cat# 74,106). Library preparation was performed using the SMARTER Stranded Total RNA Seq kit v2-Pico Input Mammalian kit (Takara Bio, cat #634411) according to the manufacturer’s instructions. Libraries were sequenced using HiSeq2500 (Illumina) at Novogene (China) to obtain at least 20 million paired-end reads per sample. Sequencing was performed in duplicates for each condition.

### mRNA-sequencing

Poly-A-enriched mRNA libraries were prepared from 1 μg total RNA using the TruSeq^®^ Stranded Library Preparation Kit (Illumina), according to the manufacturer’s instructions. mRNA-sequencing was performed for each biological duplicates of samples by Novogene (China). A total of 100 million 150 bp paired-end strand-specific reads were sequenced per sample on an Illumina HiSeq^®^ 2500 Platform (Illumina).

### Poly-ribo-sequencing

Polysome fractions were collected using the fractionation by high performance liquid chromatography-based RiboMega-SEC approach as previously described [29]. Briefly, cells were lysed in a CHAPS buffer containing RNase inhibitors and the lysate separated by size exclusion chromatography on a Thermo Dionex BioRS UHPLC and Agilent SEC-5 7.8 × 300 mm HPLC column with 2000 Å pores and 5 μm particles. The samples and column were kept at 5 °C throughout analysis. Fractions were collected across the entire separation covering polysomes, monosomes and smaller cellular complexes. Total RNA was extracted from the eluted polysome fractions using TRIzol LS (ThermoFisher, cat# 10296010). Ribosomal RNA depleted library 1 μg total RNA using the Illumina Stranded Total RNA Prep with Ribo-Zero Plus Kit (Illumina), according to the manufacturer’s instructions. A total of 25 million 150 bp paired-end strand-specific reads were sequenced per sample on an Illumina HiSeq^®^ 2500 Platform (Illumina).

### Bioinformatic analyses

For all sequencing data, the raw reads were subject to quality check using FastQC [30]. Adapter and poor-quality sequences were removed using Trimmomatic (version 0.39) with default settings [31]. Pair-end clean reads were then mapped to the human reference genome hg38 (ENSEMBL 86 release, GRCm38) using STAR (version 2.5.2a) [32]. Read counts for reads overlapping genomic regions were quantified with FeatureCounts from the Subread package [33]. For poly-A enriched mRNA-sequencing data, differentially expressed genes were determined using the R package DESeq2 (version 3.7) [34] with reads per kilo base per million (RPKM) > 1, a Benjamini-Hochberg (BH)-adjusted *P* < 0.05 and an absolute value of a fold change > 2.0. For poly-ribo sequencing data, trimmed reads were filtered for mitochondrial DNA and ribosomal RNA using Bowtie2 (version 2.3.4) [35]. Differential enrichment of mRNA transcripts in the polysome fraction was determined using DESeq2 with the same parameters used for analyzing mRNA-sequencing data.

For the analysis of m^6^A-RIP-sequencing data, only uniquely mapped genes were selected by samtools for downstream analysis. m^6^A peaks enriched in immunoprecipitation over corresponding input samples were identified by MACS2 (version 2.1.0.20150731) [36]. The effective genome size was calculated by the reference annotation. Significant (FDR-adjusted *P* < 0.05, |fold change between IP and input |≥ 2.0, and a minimum peak read count of 10 across replicates and conditions) peaks were considered.

To identify high confidence or consensus m^6^A peaks, peaks were intersected in a pairwise fashion among two replicates using the BedTools package with a setting of ‘-f 0.5’ [37]. Consensus m^6^A peaks were mapped to the coding sequences (CDS), 5′UTRs, 3′UTRs, start codon, stop codon and non-coding RNAs (ncRNAs), in that order by using intersectBed from BEDTools according to the RefSeq gene annotations (Ensembl version 86). m^6^A consensus DRACH motif were identified by performing de novo motif search with the HOMER software (version 4.9.1) [38] with m^6^A peaks as the target sequences and control peaks as the background sequences. The motif length was restricted to 5 nucleotides. The motif with most significant *P*-value was visualised using WebLogo [39]. The metagene profile was plotted with the ‘Guitar’ R package [40].m^6^A consensus peaks among control and VIRMA-Overexpressed were merged using the BedTools and bedtools multicov was used to get the count table of input and IP for each peak region. The m^6^A peaks from any two conditions were classified as unique and common based on whether they overlapped or not. DESeq2 (version 3.7) was used to test whether the m^6^A peak levels were significantly different (BH-adjust *P* < 0.05) when they show fold enrichment > 1.5.

### Functional enrichment analysis

Gene ontology (GO) enrichment analysis was performed for the genes with significantly increased/decreased genes by using ‘clusterProfiler’ package [41]. The GO enrichment was plotted using ‘ggplot2’ R package. GSEA (version 4.1.0) [42] and MSigDB Genesets (c2.all.V7.5.symbols.gmt) [43] were downloaded to perform the Gene set enrichment analysis using GSEAPreranked program. Prior to analysis, a ranked list was calculated with each gene symbol assigned a score based on the BH-adjust *P* value and the direction of the log fold-change (“increased” or “decreased”). Gene sets identified as significant (*P* < 0.05) with GSEA were visualised using the Enrichment Map plugin available for Cytoscape version 3.8.2[44].

### RNA extraction, cDNA synthesis and RT-qPCR

For RNA extraction, samples were lysed in 0.5 mL or 1 mL TRIzol Reagent for each well in 12-well or 6-well plates, respectively (Invitrogen, cat# 15596018). For *NEAT1* analysis, the lysates were further sheared up and down 30 × using 25 g needle on 1 mL syringe. 100 μL or 200 μL chloroform (Sigma, cat# C2432-500ML) was added to 0.5 mL or 1 mL TRIzol lysates respectively, shook well, and left at room temperature for 15 min for phase separation. Samples were then centrifuged at 12,000*g* for 20 min at 4 °C. The upper interface was then transferred to new 1.5 mL tubes containing 250 μL or 500 μL isopropanol (Sigma, cat# 278475-2L) containing 1–2 μL glycogen (ThermoFisher, cat# AM9510), mixed well and placed into − 30 °C freezer for 1–2 h. The RNA was pelleted at 16,000×*g* for 20 min at 4 °C, then subjected to 0.5 mL or 1 mL 75% ethanol wash and centrifuged again at 16,000*g* for 5 min at 4 °C. The pellets were air-dried, resuspended in appropriate volumes of DNase-free RNase-free water (ThermoFisher, cat# 10977023) and incubated at 60 °C for 5 min to further solubilize the RNA.

For *XBP1* splicing, the iScript gDNA clear cDNA synthesis kit (BioRad, cat# 1725035) containing both random hexamers and oligo-dT was used as per manufacturer’s instructions. Briefly, 1 μg of RNA was treated in 1 × DNase Buffer in 16 μL volume at 25 °C for 5 min, followed by heat inactivation at 75 °C for 5 min and 4 °C cooling. The RNA was then diluted 1:2 and halved evenly for RT + or RT−. 4μL of RT + or RT− supermix was added, resulting in a total volume of 20 μL. The samples were then subjected to a program of 25 °C for 5 min, 46 °C for 20 min and 95 °C for 1 min, then cooled down to 4 °C. Samples were diluted 1:2 and 2 μL were used as cDNA template for RT-qPCR.

For *NEAT1_1* measurement, cDNA synthesis with oligo-dT18 (Bioline, cat# BIO-38029) was used. 1 μg of RNA was either treated with Turbo DNA-free Kit (ThermoFisher, cat# AM1907) or with DNase I amplification grade (ThermoFisher, cat# 18068015) according to the manufacturer’s instructions. 1 μL oligo-dT and 1 μL water were added to each tube, then placed onto the thermocycler at 70 °C for 5 min and cooled at 4 °C. For a final volume of 50 μL, 2.5 μL 10 mM dNTP stock (Bioline, cat# BIO-39028, diluted 1:10 prior use), 2.5 μL 0.1 M DTT, 10 μL 5 × first strand buffer, 20 μL 5 M Betaine, 200U 1 μL SuperScript™III Reverse Transcriptase (Invitrogen, cat# 18080044) and 40U 1 μL RNaseOUT™ Recombinant Ribonuclease Inhibitor (Invitrogen, cat# 10777019) were added to the RNA for cDNA synthesis at 55 °C for 60 min, 70 °C for 15 min and then on hold at 4 °C. 2 μL was used as cDNA template for RT-qPCR.

To perform RT-qPCR, technical duplicates or triplicates were performed per RT + samples. RT− samples were also run to confirm the signals detected were not due to DNA contaminant. 8 μL of cocktail containing 5 μL 2 × SensiFAST SYBR No-ROX mastermix (Bioline, cat# BIO-98050), 0.5 μL of 6 mM forward primer, 0.5 μL of 6 mM reverse primer and 2 μL of water was added to each well in 384-well PCR plate (BioRad, cat# HSR4801). 2 μL cDNA template was then added on top for a total volume of 10 μL. The plate was then sealed with Opti-seal optical disposable adhesive (Astral Scientific, cat# 157300) and centrifuged at 1000×*g* for 5 min at 25 °C. The plate was then run on the Roche LightCycler 480 with the following conditions: Initial denaturation at 95 °C for 3 min, 40 cycles of 95 °C for 10 s, 60 °C for 30 s and 72 °C for 20 s, melt curve generation from 95 °C for 5 s, 60 °C for 1 min and then continuous increase in temperature until 95 °C was reached. Primer sequences are detailed in Table S1.

### Transfection of sno-plasmid and transfection efficiency analysis

Sno-plasmids which allowed for overexpression of long non-coding RNA, *NEAT1_1* (pZW1-sno-*NEAT1-1*) and control (pZW1-sno-vector) were kindly provided by A/Prof Archa Fox (University of Western Australia)[45]. For each replicate, 3 μg of the plasmid was transfected using X-tremeGENE-9 (Sigma, cat# 6365779001) in each well of a 6-well plate with 3.5 × 10^5^ SKBR3 cells seeded the day before. Media was changed 3–4 h post transfection and the cells were grown in the 37 °C incubator with 5% CO_2_ for 48 h. Cells were harvested for assessment of transfection efficiency, RT-qPCR and cell proliferation assay using the CCK8 kit (Abcam cat# ab228554).

For transfection efficiency analysis, cells were resuspended in FACSWash Buffer (1 × PBS containing 2.5% Fetal Bovine Serum (HyClone™, cat# SH30084.03), 2 mM EDTA (ThermoFisher, cat# AM9261), 0.01% sodium azide (Sigma, cat# 71289-5G)) containing 0.05μg/mL DAPI (4,6-diamidino-2-phenylindole dihydrochloride, Invitrogen, cat# D1306) for live/dead discrimination. Samples were acquired on LSR Fortessa (BD) to determine the percentage of GFP +ve cells and data were analysed using Flowjo X (BD).

### Immunofluorescence

Cells were plated at > 50% density onto 18 mm circular #1.5 glass coverslips and fixed after 24 h in 4% Paraformaldehyde (Sigma, cat# 158127) pH 7.4 for 15 min at 37 °C. Coverslips were then washed in PBS 3 × 10 min and permeabilised in 0.2% Triton-X for 20 min under gentle rocking. Cells were washed 3 × 10 min and blocked overnight in 5% BSA. Coverslips were treated with primary antibodies for 3 h in 5% BSA (Sigma, cat# A7030), washed in PBS 3 × 20 min, and then incubated with secondary antibodies for 1 h in PBS. Coverslips were washed 3 × 20 min, treated with ATTO488-Phalloidin (Sigma, cat# 49409) for 15 min, then DAPI 2 μg/mL for 10 min, washed 3 × 2 min, and then inverted onto 15 μL of ProLong Antifade mounting medium. Coverslips were left to cure overnight in the dark at room temperature.

A C2 Nikon Basic confocal microscope with 405, 488, 640 nm lasers was used to acquire 1.5 μm z-stacks centered on the nuclei with 3 standard photomultiplier tubes. *n* ≥ 5 fields of view were acquired with a Nikon 40 × Plan Apo 0.95 objective and condensed into maximum intensity z-projections. Nuclear and cytoplasmic fluorescence intensity were measured using standard cell segmentation tools in ImageJ/FIJI using DAPI and phalloidin signal as masks (protocol for batch processing available upon request). The ratio of nuclear to cytoplasmic fluorescent intensity was then calculated per cell.

### Hypoxia treatment

SKBR3 cells were seeded in a six-well plate to 80–90% confluency, with AeraSeal™ film covering the plate in place of a lid for more efficient gas transfer. The plate was then placed in a custom-built acrylic hypoxic chamber connected to a programmable gas blender (3-channel GB100, MCQ Instruments) within a standard humidified cell culture 37 °C incubator with 5% CO_2_ for up to 24 h. The oxygen concentration in the hypoxic gas mixer (chamber) was maintained at 95% nitrogen, < 0.05% O_2_ and 5% CO_2_ as monitored with Profiling oxygen microsensor (PM-PSt7, PreSens) connected to a compact oxygen transmitter (OXY-1 ST, PreSens) [46]. After hypoxia treatment, the existing supernatant was immediately aspirated and the cells were subjected to 1 × PBS wash and aspirated. Then 150–200 μL RIPA buffer (Sigma, cat# R0278-500ML) with 1 × Protease Inhibitor Cocktail (Sigma, cat# S8830) and 1 × PhosSTOP (Sigma, cat# 4906845001) were added to each well and cell scrapers were used to detach cells. The lysate was mixed well and placed on ice for 15–30 min, centrifuged at 16,000×*g* for 5 min at 4 °C and transferred to a new 1.5 mL tube.

### Treatment of cells with UPR-inducing agents

SKBR3, MDA-MB-231 and HS578 T were plated in 6-well plates with 80–85% confluency at the time of treatment with UPR-inducing agents, either in 2 mL of 100 nM thapsigargin (Sigma, cat# T9033-1MG) or 2 mL of 0.5 mM sodium arsenite (Sigma, cat# S7400-100G), for a specified period in 37ºC incubator with 5% CO_2_. At harvest, existing supernatant was aspirated, then cells were washed with 1 mL 1 × PBS. For protein lysate, 200 μL RIPA buffer (Sigma, cat# R0278-500ML) with 1 × Protease Inhibitor Cocktail (Sigma, cat# S8830) and 1 × PhosSTOP (Sigma, cat# 4906845001) was added to each well, with cell scrapers used to detach cells. The lysate was pipetted up and down and left on ice for 15–30 min and centrifuged at 16,000 *g* for 5 min at 4 °C. The lysate was transferred away from the pellet to a new 1.5 mL tube. For RNA, 1 mL of TRIzol Reagent (Invitrogen, cat# 15596018) was added to lyse the cells and transferred into 1.5 mL tubes.

For cell viability assessment, cells were seeded in 96-well plates for 70% confluency on the day of treatment. Thapsigargin was diluted to a range of concentrations in the corresponding media and added to the cells for 48 h. 5 μL CCK8 was added to each well and absorbance at 450 nm was measured at least 3 h or more post CCK8 addition. Absorbance values were normalised to 0 nM, expressed as percentage of viability.

### Statistical analysis

Unless stated otherwise in the text and/or figure legends, all analyses were performed using Graph-Pad Prism v.8 (La Jolla, CA, USA). Student’s *t* tests were used to determine significance unless indicated otherwise in the figure legends. *P* < 0.05 was considered significant for all tests.

## Results

### Amplification of *VIRMA* is associated with the overexpression of *VIRMA* transcripts and adverse outcome in breast cancer

**Fig. 1:**
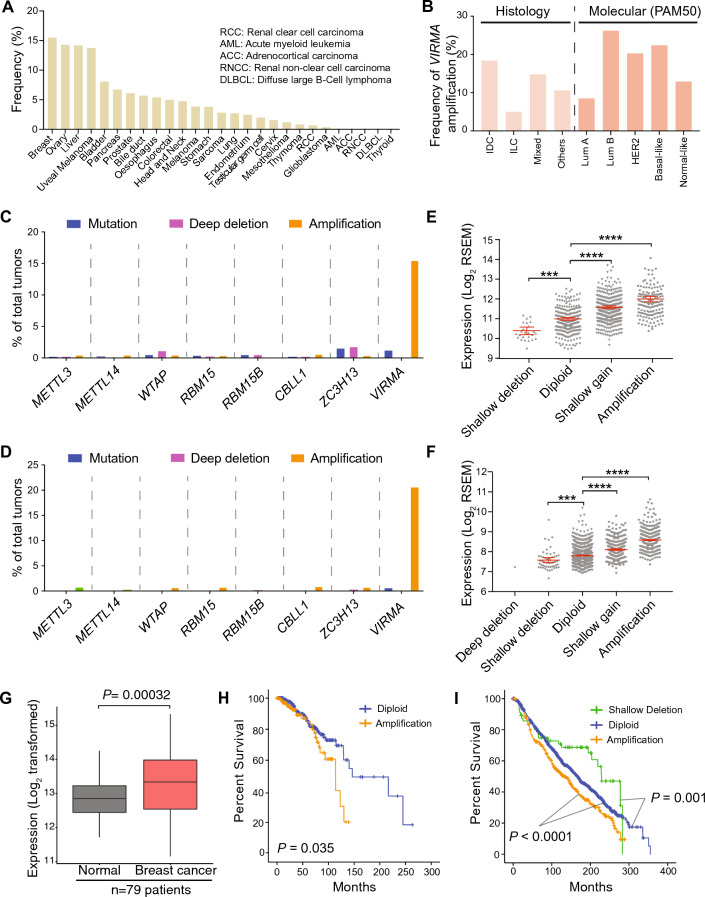
Amplification of VIRMA is frequent in breast cancer and is associated with poorer overall survival. **A** The fraction of cancers with *VIRMA* amplification within each of the 27 types of cancers from TCGA datasets. **B** Distribution of breast cancer with *VIRMA* amplification in the TCGA cohort based on histology and molecular classifications (*IDC* invasive ductal carcinoma, *ILC* invasive lobular carcinoma, *Lum* luminal). **C** The frequency of mutation and genetic alterations of genes encoding components of the m^6^A methyltransferase complex in the TCGA breast cancer cohort. **D** The frequency of mutation and genetic alterations of genes encoding components of the m^6^A methyltransferase complex from the METABRIC breast cancer cohort. **E**, **F** mRNA expression of *VIRMA* in breast cancers bearing different types of genetic alterations in the TCGA and METABRIC cohorts. **G** mRNA expression of *VIRMA* in breast cancer compared to matched normal controls from the TCGA cohort. **H** Kaplan–Meier survival plots comparing overall survival of TCGA breast cancer patients bearing tumours with amplification and those without any genetic alteration to *VIRMA*. **I** Overall survival analysis of METABRIC breast cancer patients bearing tumours with amplification, shallow deletion and no genetic alteration to *VIRMA*. *P* < 0.05 denotes significant. In **E** and **F**, significance was determined using one-way ANOVA followed by Tukey’s test for multiple comparison of the means: ***, *P* < 0.001; ****, *P* < 0.0001, error bars indicate mean ± SEM. In **G** significance was determined using unpaired two-tailed Student’s *t* test. In **H** and **I**, significance was determined using log-rank test

We focused on breast cancer as it has the highest frequency of *VIRMA* amplification among the 27 cancers in the Cancer Genome Atlas Research Network (TCGA) cohorts (Fig. 1A). Based on histological assessments, *VIRMA* amplification is more frequent in invasive ductal carcinoma (18%) compared to invasive lobular carcinoma (6%); the former is also the more common subtype of breast cancer (Figs. 1B, S1A–D). Based on molecular subtypes, *VIRMA* amplification is more common in the more aggressive Luminal B, Her2 and Basal-like subtypes (> 20% in each) compared to Luminal A (8.5%) (Figs. 1B, S2A–E). *VIRMA* is also the only component of the m^6^A methyltransferase complex that showed a high frequency of amplification in the TCGA cohort (15%, *n* = 960, Fig. 1C). We confirmed the high frequency of *VIRMA* amplification (20%) in an independent cohort from the Molecular Taxonomy of Breast Cancer International Consortium, METABRIC (*n* = 1904, Fig. 1D) [47]. Notably, tumours with *VIRMA* amplification exhibited significantly increased expression of *VIRMA* mRNA compared to samples without profound genetic alteration of *VIRMA* (Diploid) in both cohorts (Figs. 1E, F, S1E–G, S2F–J).

We further observed significantly increased expression of *VIRMA* in breast cancers compared to matched normal breast tissues available from 79 patients in the TCGA cohort (Fig. 1G). Apart from RBM15, which has been reported to recruit the core m^6^A methyltransferase to specific sites via recognition of U-rich sequences [48], none of the other components of the m^6^A methyltransferase complex show increased expression in breast cancers (Fig. S3A–G).

Of clinical importance, amplification of *VIRMA* is associated with poorer overall survival in both the TCGA and METABRIC cohorts (Fig. 1H, I). Shallow deletion of *VIRMA* is associated with better overall survival in the METABRIC cohort (Fig. 1I). Collectively, we observe a high frequency of *VIRMA* amplification, which is associated with higher expression of *VIRMA* across all subtypes of breast cancers. The association between *VIRMA* amplification status and overall survival indicates the potential utility of *VIRMA* expression/amplification status for prognostic purposes.

### Overexpression of the full-length but not the N-terminal VIRMA enhances breast cancer growth in vitro and in vivo

**Fig. 2:**
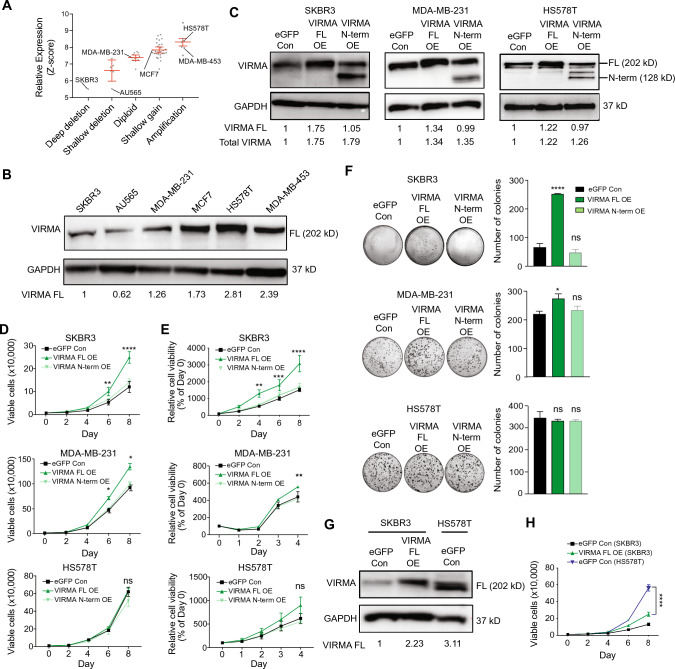
Overexpression of full-length VIRMA promotes growth of breast cancer cells in vitro. **A** Expression of *VIRMA* mRNA in breast cancer cell lines with different types of genetic alterations. Data were extracted from the Cancer Cell Line Encyclopedia (Broad Institute). **B** Representative western blot showing the endogenous levels of VIRMA protein in breast cancer cell lines. Fold-difference of VIRMA normalised to a loading control (GAPDH) is shown. **C** Representative western blots showing the expression of full-length (202 kD) and N-terminal VIRMA (128 kD) in breast cancer cell lines following lentiviral-mediated transduction of full-length (FL OE) and N-terminal (N-term OE) VIRMA compared to control cells (eGFP Con). GAPDH was included as the loading control. For each cell line, fold-change of VIRMA normalised to the loading control (GAPDH) is shown for VIRMA FL OE and N-term OE relative to eGFP control. FL, full length; N-term, N-terminal. **D** Number of viable cells counted for breast cancer cells overexpressing the full-length or N-terminal VIRMA, and control cells cultured over 8 days. Counting was performed every two days. **E** Cell growth after overexpression of full-length and N-terminal VIRMA in breast cancer cell lines analysed using the MTT or CCK8 assay. **F** The colony formation assay performed on breast cancer cell lines transduced with lentivirus expressing full-length and N-terminal VIRMA. Bar plots of the number of colonies counted for each group (*n* = 3 plates per experimental condition). Two-way ANOVA was used to determine the significance in **D**, **E** and **H**. For **D** and **E**, significance is only shown for VIRMA FL OE compared to eGFP Con. One-way ANOVA was used to determine the significance in **F**. Multiple comparisons of the means were performed using the Tukey’s test. Data are from ≥ 3 biological replicates and show mean ± SEM. *, *P* < 0.05; **, *P* < 0.01; ***, *P* < 0.001; ****,* P* < 0.0001; ns, not significant

To confirm the role of VIRMA overexpression in breast cancer, we expressed the HA-tagged full-length VIRMA (VIRMA FL) and the VIRMA N-terminal isoform (VIRMA N-term) in breast cancer cell lines bearing low (SKBR3 and AU565), intermediate (MDA-MB-231) and high (HS578T) endogenous levels of *VIRMA* (Figs. 2A–B, S4A–C). Only the full-length isoform of VIRMA is readily detected in control cells (Figs. 2B, C, S4C), indicating that VIRMA FL is the dominant isoform in breast cancer cells. In cell lines with low and intermediate endogenous levels of *VIRMA*, VIRMA FL overexpression significantly increased cell proliferation and colony formation capacity (Figs. 2D–F, S4D, S4E). No significant difference in cell proliferation and clonogenicity was observed in VIRMA FL-overexpressing HS578T cells compared to control HS578T cells overexpressing eGFP only (Fig. 2D–F). Control HS578T cells harbour endogenous expression of VIRMA at a level that is higher than that expressed in SKBR3 overexpressing VIRMA FL (Fig. 2G). Accordingly, control HS578T cells proliferate faster than SKBR3 cells overexpressing VIRMA FL (Fig. 2H). Compared to control cells, overexpression of VIRMA N-term did not alter cell proliferation and clonogenicity in all four breast cancer cell lines examined (Figs. 2D–F, S4D, S4E). Consistent with VIRMA being an essential protein for cell survival, the growth and colony-forming capacity of breast cancer cells were markedly perturbed consequent to VIRMA depletion in vitro (Fig. S4F–H).

**Fig. 3:**
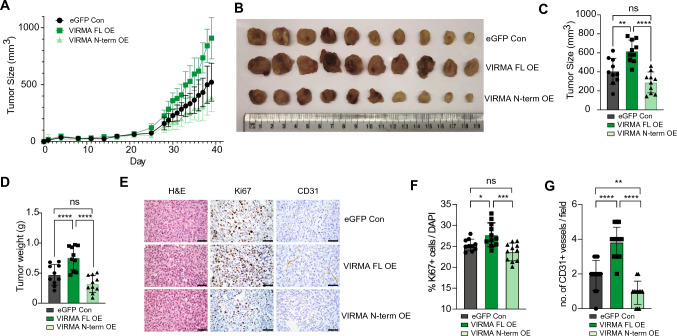
Overexpression of full-length VIRMA enhances tumour development in vivo. **A** Tumour size measured over a period of 39 days in MDA-MB-231 xenografts overexpressing two different isoforms of VIRMA and eGFP alone (*n* = 10 per group). **B** Tumours collected at endpoint for each experimental group. **C** Measurement of the size and **D** weight of the tumours harvested at endpoint. **E** Representative images showing immunohistochemistry analysis of tumours from xenografts **B** obtained at the experiment endpoint. H&E, hematoxylin and eosin. Scale bars represent 50 μm. **F** Percentage Ki67 positive cells normalised to DAPI in tumours overexpressing the two isoforms of VIRMA and eGFP control (*n* = 10 fields from three tumours per group). **G** Number of CD31 positive vessels per field (*n* ≥ 12 fields from three tumours per group). In **C**, **D**, **F** and **G**, statistical significance was determined using one-way ANOVA. Multiple comparisons of the means were performed using the Tukey’s test. Data are from ≥ 3 biological replicates and show mean ± SD. *, *P* < 0.05; **, *P* < 0.01; ***, *P* < 0.001; ****,* P* < 0.0001; *ns* not significant

Overexpression of VIRMA FL in MDA-MB-231 cells significantly enhanced their growth in immunocompromised mice compared to VIRMA N-term-overexpressing and control cells (Fig. 3A–D). The enhancement of tumour growth in VIRMA FL-overexpressing xenografts was supported by increased proliferation and angiogenesis as indicated by increased Ki67 and CD31 levels in the tumours, respectively (Fig. 3E–G). Overall, our results demonstrate that overexpression of full-length VIRMA is necessary to enhance growth and colony formation of breast cancer cells in vitro and in vivo.

### Full-length but not the N-terminal VIRMA regulates m^6^A methylation

**Fig. 4:**
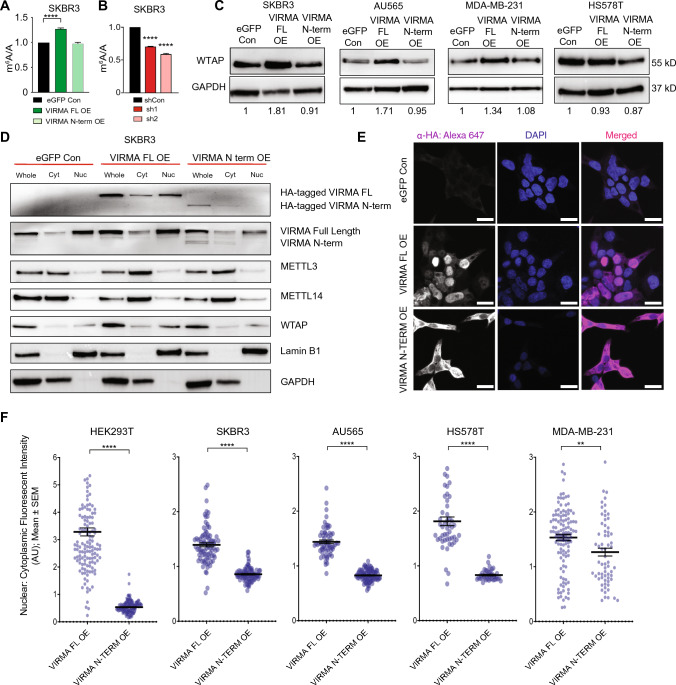
Overexpression of the nuclear-enriched full-length VIRMA but not the cytoplasmic-localised N-terminal VIRMA increases m^6^A RNA methylation. **A** m^6^A/A ratio on polyadenylated RNAs from SKBR3 cells transduced with lentivirus expressing full-length (FL OE) or N-terminal (N-term OE) VIRMA compared to control cells (eGFP Con). **B** m^6^A/A ratio on polyadenylated RNAs from SKBR3 cells transduced with lentivirus expressing shRNAs (sh1 and sh2) against VIRMA and a non-targeting control (shCon). In **A** and **B**, statistical significance was determined using one-way ANOVA with Tukey’s multiple comparisons of the means. **C** Representative western blots of WTAP expression in breast cancer cell lines following lentiviral-mediated transduction of full-length and N-terminal VIRMA compared to controls. For each cell line, fold-change of WTAP normalised to the loading control (GAPDH) is shown for VIRMA FL OE and N-term OE relative to eGFP control. **D** Representative western blots of VIRMA, METTL3, METTL14 and WTAP levels in the whole cell (Whole), nuclear (Nuc) and cytoplasmic (Cyt) fractions obtained from SKBR3 cells transduced with lentivirus overexpressing full-length VIRMA, N-terminal VIRMA and eGFP control. Lamin B1 and GAPDH were included to confirm the purity of nuclear and cytoplasmic extract respectively. **E** Immunofluorescence imaging of HA-tagged full-length or N-terminal VIRMA conjugated to Alexa 647 in HEK293T cells. DAPI was used as the positive control for nuclear staining. Scale bars represent 50 μm. **F** Quantification of nuclear:cytoplasmic fluorescent intensity of Alexa 647 positive cells in HEK293T and breast cancer cell lines transduced with lentivirus expressing full-length or N-terminal VIRMA. Statistical significance was determined using unpaired two-tailed Student’s *t* test. Data are from ≥ 3 biological replicates and show mean ± SEM. **, *P* < 0.01; ****, *P* < 0.0001

To determine the link between VIRMA levels and m^6^A changes, we assessed m^6^A levels in polyadenylated RNAs in VIRMA-overexpressing SKBR3 cells using mass spectrometry (LC–MS/MS). Notably, overexpression of VIRMA FL but not the VIRMA N-term significantly increased overall m^6^A levels (Fig. 4A). shRNA-mediated knockdown of VIRMA in SKBR3 and other cell lines, HS578T, MCF7 and MDA-MB-453 led to a significant decrease in m^6^A levels (Fig. 4B, S4I).

Increased expression of METTL3 has previously been shown to upregulate the expression of WTAP in an m^6^A-dependent manner [49]. We therefore examined whether overexpression of VIRMA affects the expression of core m^6^A methyltransferases. While we did not observe obvious changes in the expression of METTL3 and METTL14 in the VIRMA FL-overexpressing cells, elevated levels of WTAP were detected in SKBR3, MDA-MB-231 and AU565 cells (Fig. 4C, S4J). No increases in METTL3, METTL14 and WTAP protein levels were detected in VIRMA FL-transduced HS578T cells that already express high levels of endogenous full-length VIRMA (Fig. 2B) or in breast cancer cells overexpressing VIRMA N-term compared to control cells (Fig. 4C, S4J). As WTAP is known to recruit and enhance the mRNA binding capability of METTL3[50], an increase in WTAP consequent to VIRMA FL overexpression is likely to enhance the function of METTL3, thereby increasing global m^6^A levels.

Given that only VIRMA FL contributed to the phenotypic and m^6^A level changes in breast cancer cells, we determined whether VIRMA FL but not VIRMA N-term is enriched in the nucleus since this is where m^6^A methylation typically occurs[50, 51]. Indeed, we found that VIRMA FL is predominantly localised to the nucleus, similar to WTAP, whereas VIRMA N-term is enriched in the cytoplasm (Fig. 4D–F, S5A–C). These results indicate that nuclear-enriched full-length but not N-terminal VIRMA regulates m^6^A changes in breast cancer cells in vitro to promote their proliferation and clonogenicity (Fig. 2D–F, S4C–E).

### Overexpression of VIRMA contributes to breast cancer growth via the m^6^A-methylated long non-coding RNA, ***NEAT1***

**Fig. 5:**
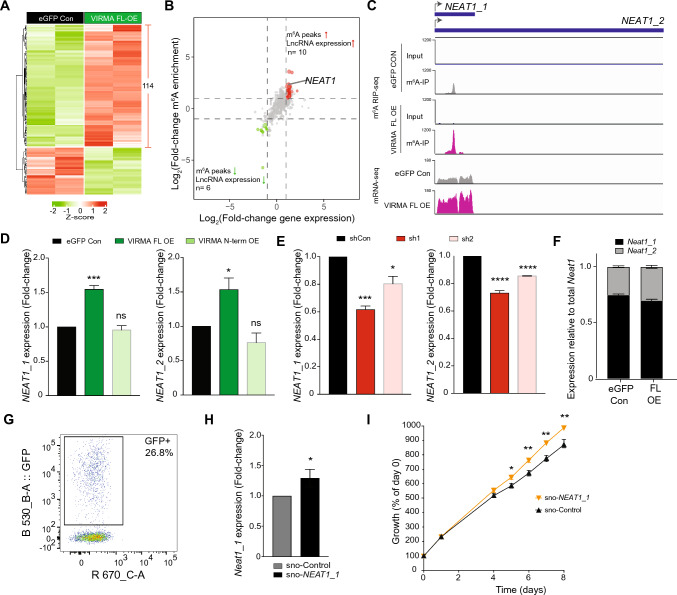
Overexpression of the full-length VIRMA enhances the expression of the long non-coding RNA *NEAT1*. **A** Heatmap showing differential expression of long non-coding RNAs (lncRNAs) in VIRMA-overexpressing SKBR3 (FL OE) and control cells (eGFP Con). **B** Differential m^6^A peak enrichment within lncRNAs in SKBR3 cells overexpressing full-length VIRMA and eGFP alone that correlates with differential expression of lncRNAs between these cells. **C** Integrative genome viewer plots showing m^6^A peaks on *NEAT1* and corresponding *NEAT1* expression in full-length VIRMA-overexpressing (magenta) and control cells (grey). Only the polyA-tailed *NEAT1_1* is visible because our polyA-enriched mRNA-seq cannot detect the non-polyA-tailed *NEAT1_2*. **D** Relative expression of *NEAT1_1* and *NEAT1_2* in SKBR3 cells overexpressing full-length or N-terminal VIRMA. **E** Relative expression of *NEAT1_1* and *NEAT1_2* in SKBR3 cells following shRNA-mediated depletion of VIRMA (sh1 and sh2) compared to a non-targeting shRNA control (shCon). **F** The percentages of *NEAT1_1* and *NEAT1_2* expressed in SKBR3 cells. **G** A representative flow cytometry plot showing percentage of GFP positive cells after transfection of SKBR3 cells with a snoRNA vector co-expressing GFP and *NEAT1_1* (sno-*NEAT1_1*). **H** Relative expression of *NEAT1_1* in SKBR3 cells after transfection with sno-*NEAT_1* compared to an empty vector control (sno-control). **I** Growth of SKBR3 cells after transfection with sno-*NEAT_1* compared to sno-control determined by the CCK8 assay. In **D** and **E**, statistical significance compared to control was determined using one-way ANOVA. In **H**, statistical significance was determined using unpaired two-tailed Student’s *t* test. Two-way ANOVA was used to determine the significance in **I**. Multiple comparisons of the means were performed using the Tukey’s test. Data are from ≥ 3 biological replicates and show mean ± SEM. *,* P* < 0.05; **, *P* < 0.01; ***, *P* < 0.001; ****, *P* < 0.0001; ns, not significant

To identify the m^6^A-regulated long non-coding RNA (lncRNA) and mRNA targets affected by VIRMA FL overexpression, we compared m^6^A peak abundance in SKBR3 cells overexpressing VIRMA FL and control via m^6^A RNA immunoprecipitation sequencing. Consistent with previous reports [52–54], we observed significant enrichment of m^6^A peaks over input control within the DRACH consensus motif near the 3' UTR and stop codons (Fig. S6A and S6B). Of the 114 lncRNAs that significantly increased in expression following VIRMA FL overexpression (*P* < 0.05, fold-change > 2, Fig. 5A and Table S2), 10 also showed enriched m^6^A peaks (*P* < 0.05, fold-change > 2, Fig. 5B and Table S3). The most notable of these lncRNAs is *NEAT1* (Fig. 5B, C), which has been reported to confer oncogenic potential in breast cancer [55–58]. Through VIRMA FL overexpression and knockdown experiments, we confirmed that increased VIRMA FL promotes the expression of both *NEAT1_1* (3.7 kb) and *NEAT1_2* (23 kb) isoforms in SKBR3 cells (Fig. 5D, E) [59]. Notably, *NEAT1_1* comprised 75% of the total *NEAT1* transcripts in wild-type SKBR3 (Fig. 5F) indicating that *NEAT1_1* is the dominant isoform in SKBR3 cells. Using a previously published sno-vector [45], we only achieved 15–27% transfection efficiency and 0.3-fold increase in *NEAT1_1* expression (Fig. 5G, H). Nevertheless, this slight upregulation in *NEAT1_1* (Fig. 5H) was sufficient to increase the proliferation of SKBR3 cells (Fig. 5I). This result is congruent with the effect of overexpressing full-length VIRMA in these cells (Fig. 2D–F). Thus, our results indicate that increased expression of VIRMA may promote the expression of m^6^A-methylated *NEAT1* that in turn triggers oncogenicity of breast cancer cells in vitro*.*

### Overexpression of VIRMA is associated with increased expression of m^6^A-methylated mRNAs that regulate the unfolded protein response

**Fig. 6:**
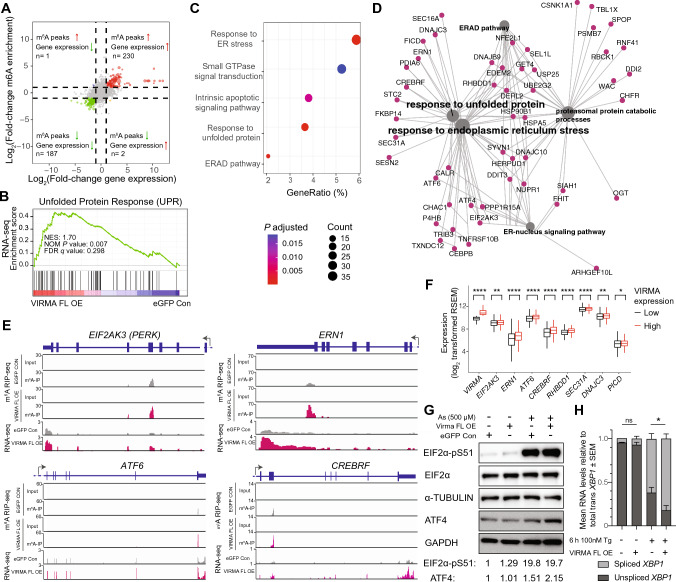
Overexpression of the full-length VIRMA enhances the levels of mRNAs involved in unfolded protein response. **A** Correlation of differential m^6^A peak enrichment in SKBR3 cells overexpressing full-length VIRMA and eGFP alone with differential mRNA expression between these cells. **B** Gene set enrichment analysis showing the enrichment score of unfolded protein response (UPR) in SKBR3 cells overexpressing full-length VIRMA. **C** Gene ontology category analysis of the pathways that significantly changed with full-length VIRMA overexpression in SKBR3 cells (*P* adjusted values with Benjamini–Hochberg correction < 0.05). Count indicates the number of significantly increased genes within a specific gene ontology category. GeneRatio (%) is the percentage of Count/total number of significantly increased genes. **D** Network map generated by Cytoscape showing genes within specific pathways that are significantly enriched in full-length VIRMA-overexpressing SKBR3 cells. **E** Integrative Genome Viewer plots showing m^6^A peaks on specific genes encoding UPR regulators and corresponding mRNA expression data in full-length VIRMA-overexpressing (magenta) and control SKBR3 cells (grey). Peaks from input controls for m^6^A-RIP-sequencing are displayed in blue. **F** The mRNA expression of genes encoding UPR regulators in TCGA breast cancers expressing high (> median) and low (< median) levels of VIRMA. **G** Western blots showing the levels of UPR proteins in VIRMA-overexpressing SKBR3 cells at baseline and after stimulation with 500 μM sodium arsenite (As) for 1 h. EIF2α and α-Tubulin were included as loading controls for EIF2α-pS51. GAPDH is the loading control for ATF4. Relative fold-change of these proteins normalised to respective loading control (EIF2α or GAPDH) is shown. **H** The proportion of spliced versus unspliced *XBP1* transcripts in VIRMA-overexpressing SKBR3 cells at baseline and after stimulation with 100 nM Thapsigargin for 6 h (Tg). In **F**, statistical significance shown was determined using unpaired two-tailed Student’s *t* test. In **H** statistical significance was determined using two-way ANOVA with multiple comparison of the means performed using the Tukey’s test. Data are from 3 biological replicates and show mean ± SEM. *,* P* < 0.05; **, *P* < 0.01; ****, *P* < 0.0001

On mRNAs, 655 m^6^A peaks were significantly increased following VIRMA FL overexpression in SKBR3 cells (*P* < 0.05; fold-change > 2, Fig. S6C, and Table S4). Increased m^6^A peaks were associated with increased mRNA expression (*P* < 0.001, Fisher’s exact test, Fig. 6A), consistent with recent reports that m^6^A methylation is coupled to active transcriptional processes [60–64].

Gene set enrichment and gene ontology analyses consistently identified UPR and related pathways as major signaling pathways affected by the overexpression of VIRMA FL in SKBR3 cells (Fig. 6B–D and Table S5). UPR, which can be triggered by ER stress, is critical for tumour growth, adaptation and response to chemotherapy but its effect in the presence of VIRMA overexpression in cancer is elusive [65]. Over a dozen mRNAs that encode UPR-related proteins including the three major molecular sensors of UPR, *EIF2AK3*, *ERN1* and *ATF6*, showed enrichment of m^6^A peaks in their UTRs and within exons (Figs. 6D, E, S6D). Expression of these mRNAs was also increased in VIRMA FL-overexpressing SKBR3 cells (Figs. 6E, S6D, S6E). Consistent with this result, analysis of the mRNA-sequencing data from the TCGA breast cancer cohort revealed significant increases in the mRNA expression of over half a dozen UPR signaling genes in samples with higher (> median) than those with lower VIRMA levels (< median) (Fig. 6F).

### Overexpression of VIRMA does not impact the translation of most m^6^A-regulated transcripts in breast cancer cells under optimal culture conditions

We then investigated whether increased VIRMA FL overexpression promotes the translation of commonly studied UPR regulators downstream of EIF2AK3; however, there were minimal changes in EIF2α-pS51 and ATF4 levels under optimal culture conditions (Fig. 6G). Similarly, no changes were observed in the abundance of the spliced *XBP1* isoform that regulates UPR via an alternative pathway downstream of ERN1 (Fig. 6H). Further confirmation using ribosome profiling indicated no drastic mRNA translation changes between VIRMA FL-overexpressing and control cells (Fig. S6F and S6G). These results indicate that the general increase in m^6^A-associated UPR gene expression consequent to VIRMA FL overexpression has little impact on overall UPR protein levels.

### VIRMA overexpression enhances the unfolded protein response to induce death of breast cancer cells

It is intriguing that VIRMA overexpression is associated with increased expression of transcripts encoding key proteins in the UPR and ER stress response pathways but does not affect UPR protein levels. Notably, we saw significantly increased levels of ATF4 and spliced *XBP1* in VIRMA FL overexpressing cells following arsenite- or Thapsigargin-induced UPR and ER stress (Fig. 6G, H). We hypothesise that the increased expression of UPR-associated transcripts in VIRMA FL overexpressing cells primed them to respond more rapidly and/or profoundly to subsequent signals that impact UPR and ER stress response. Understanding this effect is clinically relevant as UPR is commonly triggered during cancer progression and tumour microenvironment remodeling induced by hypoxia and in response to therapy.

**Fig. 7:**
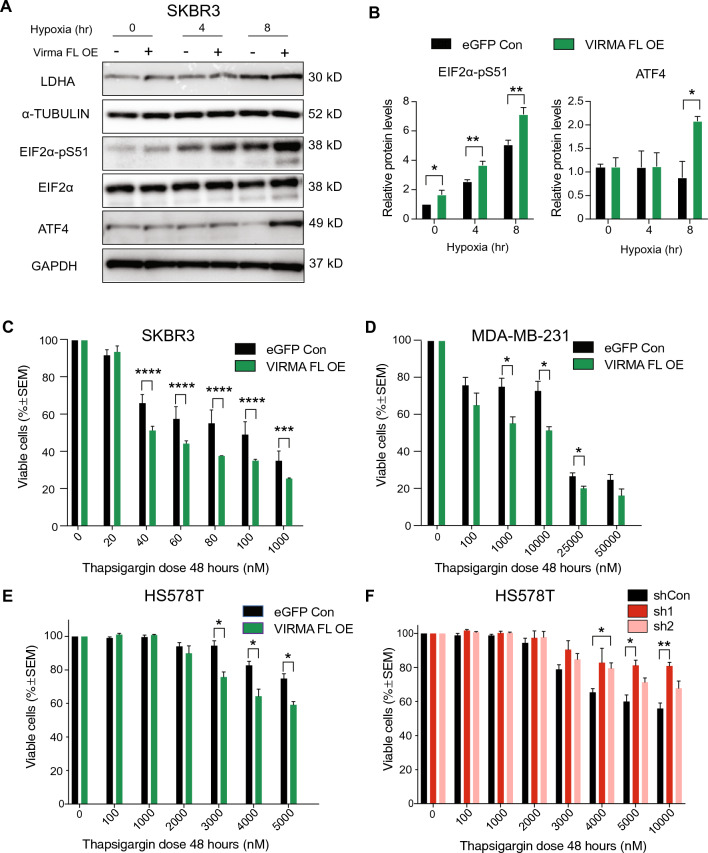
Overexpression of the full-length VIRMA enhances UPR signaling and susceptibility to cell death in breast cancer cells in vitro. **A** Representative western blots showing the levels of UPR proteins in SKBR3 cells transduced with lentivirus expressing full-length VIRMA (FL OE) and eGFP alone (eGFP Con) at baseline and after exposure to 0.05% oxygen (hypoxia) for 4, 8 and 24 h. Increased LDHA levels confirmed the induction of hypoxia. α-Tubulin and EIF2α were included as loading controls for LDHA and EIF2α-pS51 respectively. GAPDH is the loading control for ATF4. **B** Relative expression of EIF2α-pS51 and ATF4 protein in full-length VIRMA overexpressing SKBR3 and control cells under the hypoxic condition described in **A**. **C**–**E** Viability of cancer cells transduced with lentivirus expressing full-length VIRMA and eGFP control upon exposure to varying doses of Thapsigargin for 48 h. **F** Viability of HS578T cells transduced with lentivirus expressing shRNAs against VIRMA (sh1 and sh2) and a non-targeting control (shCon) upon exposure to varying doses of Thapsigargin for 48 h. All statistical significance shown was determined using two-way ANOVA with multiple comparison of the means performed using the Tukey’s test. *,* P* < 0.05; **, *P* < 0.01; ***, *P* < 0.001; ****, *P* < 0.0001; *ns* not significant. Data are from ≥ 3 biological replicates and show mean ± SEM

In a time-course assay, we confirmed that UPR and ER response proteins, EIF2α-pS51 and ATF4, increased in SKBR3 cells overexpressing full-length VIRMA compared to control cells under hypoxic conditions (< 0.05% O_2_) (Fig. 7A, B). We were unable to achieve extreme hypoxia (< 0.02% O_2_) required for UPR enhancement in MDA-MB-231 and HS578T cells. However, our results indicated increased EIF2α-pS51 and ATF4 in VIRMA FL-overexpressing MDA-MB-231 and HS578T cells when ER stress was enhanced via treatment with sodium arsenite (Fig. S7A-D) or Thapsigargin (Fig. S7E). A significantly higher proportion of spliced *XBP1* transcripts were also observed in VIRMA FL-overexpressing than control cells following treatment with Thapsigargin (Fig. S7F). Overall, we provide evidence that stressful environment can trigger the enhancement of UPR and ER stress signalling in VIRMA FL-overexpressing breast cancer cells in vitro.

UPR has been shown to maintain tumour survival and resistance to chemotherapy but sustained UPR activation could render tumours vulnerable to cell death [66–68]. To determine the effect of VIRMA overexpression on cancer cell survival following enhanced UPR activation and ER stress, we compared the viability of VIRMA FL-overexpressing and control cells exposed to increasing doses of Thapsigargin for 48 h. In all cell lines tested, we saw significantly increased sensitivity of VIRMA FL-overexpressing cells to Thapsigargin (Fig. 7C–E). Notably, knockdown of VIRMA increased cell viability in the presence of UPR and ER stress (Fig. 7F). Our results suggest that VIRMA overexpression sensitizes breast cancer cells to induce death via enhanced UPR and ER stress, while the opposite effect was observed with VIRMA depletion.

## Discussion

Despite a wealth of recent reports that describe the roles of m^6^A RNA methylation in cancers, most studies have linked them to the aberrant expression of core m^6^A writers, erasers and readers. The mechanisms by which aberrant expression of auxiliary components of the m^6^A writers complex promote tumourigenesis and the potential of exploiting these changes for therapy are underappreciated. Our focus on VIRMA, a scaffolding protein that stabilizes the m^6^A writer complex, has yielded substantial evidence that aberrant expression of an auxiliary m^6^A writer protein may affect tumourigenesis and cancer cell survival.

Besides confirming recent reports that VIRMA overexpression promotes breast tumourigenesis and confers poorer overall survival [17, 19], we reveal that VIRMA amplification affects all subtypes of breast cancer. Our results demonstrate that VIRMA has a role in pathologically relevant m^6^A changes and indicate the involvement of the full-length but not the N-terminal VIRMA isoform. By confirming that full-length VIRMA is enriched in the nucleus where m^6^A deposition occurs, while N-terminal VIRMA is generally cytoplasmic, our work explains why the previously reported role of N-terminal VIRMA in breast cancer is m^6^A-independent [17]. However, as full-length VIRMA is the dominant isoform in breast cancers (Fig. 2B, S4C), its overexpression and impact on m^6^A changes is likely to be more relevant to the development and maintenance of breast cancer than N-terminal VIRMA. Our finding that full-length VIRMA overexpression increased WTAP expression provides a possible explanation for the consequent increase in m^6^A levels on polyadenylated RNAs, consistent with a previous report that WTAP anchors METTL3/14 to mRNAs to allow m^6^A deposition [50]. However, full-length VIRMA overexpression has minimal impact on the expression of other core components of the m^6^A-methyltransferase complex (Figs. S3, S4J), indicating the possibility that full-length VIRMA has other m^6^A-independent role in breast cancer. This possibility warrants further investigation.

Multiple studies have reported the role of m^6^A in regulating oncogenic pathways in breast cancer including Wnt signaling, cell cycle deregulation, apoptosis and cancer stem cell development by altering the expression of coding and non-coding RNAs [69–73]. In the present study, we have identified a previously unreported contribution of the lncRNA, *NEAT1*, to cancer cell proliferation via VIRMA-mediated m^6^A methylation. Both the *NEAT1_1* and *NEAT1_2* isoforms have previously been shown to promote cancer cell growth [45, 55, 56]. Due to technical difficulties in overexpressing the 23 kb long *NEAT1_2*, we only confirmed the role of the shorter *NEAT1_1* isoform in promoting cancer cell proliferation in vitro (Fig. 5H, I). Moreover, *NEAT1_1* isoform may have a greater contribution to cancer growth because it constitutes up to three-quarters of total *NEAT_1* expressed in breast cancer cells (Fig. 5F). A recent study has reported that *NEAT1_1* forms a scaffold bridge to promote the assembly of PGK1/PGAM1/ENO1 complexes and enhances glycolysis [56]. This process is essential for the growth and progression of breast cancer [56]. Notably, *NEAT1_1* together with the PGK1/PGAM1/ENO1 complexes need to be exported to the cytoplasm for enhanced glycolysis to occur [56]. Given that m^6^A regulates nuclear export [74], it would be interesting to determine whether increased m^6^A deposition on *NEAT1_1* also contributes to this process.

While our study identified the UPR/ER stress pathway as being the most up-regulated consequent to VIRMA overexpression, the increase in m^6^A on transcripts encoding UPR proteins did not readily contribute to enhanced translation of proteins in this pathway. Our data suggest that m^6^A may be priming transcripts encoding UPR proteins to undergo more rapid and efficient translation in response to stress signals that are typically present in the tumour microenvironment. More importantly, the increased sensitivity of full-length VIRMA-overexpressing cancer cells to prolonged UPR presents a vulnerability that may be exploited for therapy (Fig. 7C–F). The proteosome inhibitor, bortezomib, the taxane-containing compound, paclitaxel, and the inhibitor of endoplasmic-reticulum-associated protein degradation (ERAD) pathway, eayarestatin, have all been reported to enhance the UPR in breast cancer [75–77]. Based on our results, a logical next step is to determine whether VIRMA-overexpression sensitizes breast cancer cells to these compounds. It is intriguing to speculate that lower doses of these compounds may be sufficient to kill VIRMA-overexpressing cancer cells, thereby minimizing drug toxicity. While pre-clinical evaluation would be required to progress our discovery into clinical trials, we present here a potential novel strategy for treatment of one-sixth of breast cancer patients bearing amplification and overexpression of VIRMA in their tumours.

In summary, we have uncovered the m^6^A-associated role of VIRMA in breast tumourigenesis via the long non-coding RNA, *NEAT1* and have shed light on the potential role of VIRMA in determining the fate of cancer cells under stress. Targeting VIRMA with inhibitors is unlikely to be feasible because VIRMA is essential for the survival of all cells. Thus, the unexpected discovery that VIRMA-overexpressing cancer cells are more sensitive to UPR presents a previously undescribed “Achilles Heel” of breast cancer subtypes that may be useful for the development of precision intervention.

### Supplementary Information

Below is the link to the electronic supplementary material.

Table S1. Oligonucleotide sequences (DOCX 14 KB)

Table S2. Long non-coding RNAs that increased in expression after lentiviral-mediated overexpression of full-length VIRMA in SKBR3 cells (XLS 24 KB)

Table S3. Long non-coding RNAs with significantly increased expression and enrichment of m^6^A peaks in SKBR3 cells overexpressing full-length VIRMA (XLSX 15 KB)

Table S4. mRNA targets with increased and decreased m^6^A peaks in SKBR3 cells overexpressing full-length VIRMA (XLSX 147 KB)

Table S5. Significantly enriched gene ontology associated with increased m^6^A peaks after overexpression of full-length VIRMA in SKBR3 cells (XLSX 14 KB)

Fig. S1. Alterations of genes encoding components of the m^6^A writer complex in histologically distinct breast cancers. (A-D) The frequency of mutation and genetic alterations of genes encoding components of the m^6^A methyltransferase complex in TCGA breast cancers with distinct histological characteristics. (E-G) mRNA expression of VIRMA in breast cancers from the TCGA cohort bearing different genetic alterations of VIRMA stratified based on histological subgroups. All statistical significance shown was determined using one-way ANOVA with Tukey’s test for multiple comparison of the means. **, *P *<0.01; ***, *P* <0.001; ****, *P* <0.0001; ns, not significant. Error bars indicate mean±SEM. (PDF 463 KB)

Fig. S2. Alterations of genes encoding components of the m^6^A writer complex in breast cancers with distinct molecular characteristics. (A–E) The frequency of mutation and genetic alterations of genes encoding components of the m^6^A methyltransferase complex in TCGA breast cancers with distinct molecular characteristics. (F-J) mRNA expression of VIRMA in TCGA breast cancers with different genetic alterations of VIRMA stratified based on molecular subgroups. All statistical significance shown was determined using one-way ANOVA with Tukey’s test for multiple comparison of the means. *, *P* <0.05; **, *P* <0.01; ***, *P* <0.001; ****, *P* <0.0001; ns, not significant. Error bars indicate mean±SEM. (PDF 493 KB)

Fig. S3. Expression of genes encoding components of the m^6^A writer complex in breast cancers and matched normal controls. (A) METTL3. (B) METTL14. (C) WTAP. (D) RBM15. (E) RBM15B. (F) CBLL1. (G) ZC3H13. A total of 79 matched tumours and normal breast tissues were included in this analysis. Statistical significance was determined using unpaired two-tailed Student’s t-test with *P* <0.05 denoting significance. (PDF 384 KB)

Fig. S4. The effects of VIRMA overexpression and knockdown in breast cancer cell lines. (A) Schematic of lentiviral vector constructs used to induce overexpression of full-length and N-terminal VIRMA in vitro. (B) Western blots showing the detection of HA-tagged full-length (FL OE) and N-terminal VIRMA (N-term OE) in transduced breast cancer cell lines. (C) Representative western blots of full-length (202 kD) and N-terminal VIRMA (128 kD) expression in transduced AU565 breast cancer cell line. FL, full length; N-term, N-terminal. Fold-difference of VIRMA normalised to a loading control (GAPDH) is shown for VIRMA FL OE and N-term OE relative to eGFP control (eGFP Con). (D) Number of viable AU565 breast cancer cells overexpressing full-length VIRMA, N-terminal VIRMA and eGFP alone (eGFP Con) cultured over 8 days. Counting was performed every two days. (E) The colony formation assay performed on AU565 breast cancer cells transduced with lentivirus expressing the full-length or N-terminal VIRMA compared to control cells. Bar plots showing the number of colonies counted for each group (n=3 plates per experimental condition). (F) Representative western blots showing the levels of VIRMA in SKBR3, HS578T, MCF7 and MDA-MB-453 breast cancer cells following lentiviral-mediated transduction with shRNAs against VIRMA (sh1 and sh2) compared to a non-targeting control (shCon). Fold-difference of VIRMA normalised to a loading control (GAPDH) is shown for VIRMA sh1 and sh2 relative to shCon. (G) Cell growth after shRNA-mediated depletion of VIRMA in breast cancer cell lines using the MTT assay. (H) Colony formation in breast cancer cell lines following VIRMA depletion (sh1 and sh2). Bar plots showing the number of colonies counted for each group (n=3 plates per experimental condition). (I) m^6^A/A ratio on polyadenylated RNAs from HS578T, MCF7 and MDA-MB-453 cells transduced with lentivirus expressing shRNAs (sh1 and sh2) against VIRMA compared to control (shCon). (J) Representative western blots showing METTL3 and METTL14 levels in breast cancer cell lines transduced with lentivirus expressing full-length VIRMA, N-terminal VIRMA and eGFP alone. Fold-difference normalised to the loading control (GAPDH) is shown for VIRMA FL OE and N-term OE relative to eGFP control. Two-way ANOVA was used to determine the significance in (D) and (G). In (E), (H) and (I), statistical significance was determined using one-way ANOVA. Multiple comparisons of the means were performed using the Tukey’s test. In (D), significance is only shown for VIRMA FL OE compared to eGFP Con. In (G), significance is shown for both sh1 (red) and sh2 (pink) compared to shCon. *, *P* <0.05; **, *P* <0.01; ***, *P* <0.001; ****, *P* <0.0001; ns, not significant. For bar plots, data are from 3 biological replicates and show mean±SEM. (PDF 6501 KB)

Fig. S5. Distinct localization of full-length and N-terminal VIRMA in cellular compartments. Representative western blot showing the enrichment of VIRMA, METTL3, METTL14 and WTAP in the whole cell (Whole), nuclear (Nuc) and cytoplasmic (Cyt) fractions obtained from (A) AU565, (B) MDA-MB-231 and (C) HS578T cells transduced with lentivirus overexpressing full-length (FL OE) or N-terminal (N-term OE) VIRMA or control (eGFP Con). Lamin B1 and GAPDH were included to confirm the purity of nuclear and cytoplasmic extract respectively. (PDF 2116 KB)

Fig. S6. The association between full-length VIRMA overexpression and unfolded protein response. (A) Metagene plots showing locus- and motif-specific enrichment of m^6^A peaks detected using m^6^A-RIP-sequencing in SKBR3 cells overexpressing full-length VIRMA (FL OE) and control cells (eGFP Con). (B) Distribution of m^6^A peaks at different genomic loci in SKBR3 cells overexpressing full-length VIRMA and control cells. (C) Distribution of increased and decreased m^6^A peaks following overexpression of full-length VIRMA in SKBR3 cells. Significantly increased or decreased peaks are represented in blue and black, respectively (log2 fold-change m^6^A enrichment > or < 2, P adjusted by Benjamini–Hochberg correction <0.05). (D) Integrative genome viewer plots showing examples of genes encoding unfolded protein response regulators with differential enrichment of m^6^A and mRNA expression following overexpression of full-length VIRMA (FL OE) in SKBR3 cells. (E) mRNA expression of UPR-related genes in VIRMA FL OE and control (eGFP Con) measured by mRNA-seq. Statistical significance was determined using unpaired two-tailed Student’s t-test. Data are from 3 biological replicates and show mean±SEM. *, *P* <0.05; **, *P* <0.01; ***, *P* <0.001; ****, *P* <0.0001. (F) Ribosome profile of SKBR3 cells transduced with lentivirus overexpressing full-length VIRMA and eGFP alone (eGFP Con). (G) Volcano plots showing differentially enriched genes in the polysome fractions following the overexpression of full-length VIRMA in SKBR3 cells compared to control. Genes with m^6^A methylation are highlighted in red. (PDF 2305 KB)

Fig. S7. Enhancement of UPR signaling under stress in MDA-MB-231 and HS578T cells consequent to full-length VIRMA overexpression. (A) Representative western blots showing the levels of UPR proteins in MDA-MB-231 cells transduced with lentivirus expressing full-length VIRMA at baseline and after exposure to sodium arsenite. (B) Relative levels of EIF2α-pS51 and ATF4 proteins in full-length VIRMA overexpressing MDA-MB231 and control cells exposed to sodium arsenite. Representative western blots as in (A) performed for HS578T cells. (D) Relative levels of EIF2α-pS51 and ATF4 proteins in full-length VIRMA overexpressing HS578T and control cells exposed to sodium arsenite. (E) Western blots showing the levels of UPR proteins in VIRMA-overexpressing MDA-MB-231 and HS578T cells at baseline and after stimulation with 3 μM Thapsigargin (Tg) for 6 hours. Fold-change of these proteins normalised to respective loading control (EIF2α or GAPDH) is shown. (F) The proportion of spliced versus unspliced XBP1 transcripts in full-length VIRMA-overexpressing MDA-MB-231 and HS578T cells at baseline and after stimulation with Thapsigargin (Tg). For bar plots, data are from 3 biological replicates and show mean±SEM. All statistical significance shown was determined using two-way ANOVA with multiple comparisons of the means performed using the Tukey’s test. *, *P* <0.05; **, *P* <0.01; ns, not significant (PDF 1245 KB)

Supplementary Methods (DOCX 17 KB)

## Data Availability

mRNA-, m^6^A-RIP- and poly-Ribo-sequencing data that support the findings from this study have been deposited in Gene Expression Omnibus under the accession numbers GSE205855, GSE205892 and GSE205873. All other datasets generated during and/or analysed during the current study are available from the corresponding author on reasonable request.
