# Supplementary material for: Overexpression of VIRMA confers vulnerability to breast cancers via the m6A-dependent regulation of unfolded protein response
Source: Cell Mol Life Sci. 2023 May 19;80(6):157. doi: 10.1007/s00018-023-04799-4 (PMC10198946; doi:10.1007/s00018-023-04799-4)
Supplement: Supplementary file 1 — Table S1. Oligonucleotide sequences (DOCX 14 KB) [file 18_2023_4799_MOESM1_ESM.docx]

**Table S1. Oligonucleotide sequences**

| **RT-qPCR Primers** | | |
| --- | --- | --- |
| **cDNA target** | **Primer** | **Sequence (5'-3')** |
| unspliced *XBP-1* | Forward | CAGACTACGTGCACCTCTGC |
|  | Reverse | CTGGGTCCAAGTTGTCCAGAAT |
| spliced *XBP-1* | Forward | GCTGAGTCCGCAGCAGGT |
|  | Reverse | CTGGGTCCAAGTTGTCCAGAAT |
| total *XBP-1* | Forward | TGAAAAACAGAGTAGCAGCTCAGA |
|  | Reverse | CCCAAGCGCTGTCTTAACTC |
| *NEAT1_1* | Forward | AAACGCTGGGAGGGTACAAG |
|  | Reverse | ATGCCCAAACTAGACCTGCC |
| *NEAT1_2* | Forward | ACAGCATTCCTGTCTGCGAA |
|  | Reverse | GACTTCAGGCTCCAGCCATT |
| *B2M* | Forward | TGCCGTGTGAACCATGTGAC |
|  | Reverse | CGGCATCTTCAAACCTCCAT |

| **shRNA sequence (5'-3')** | |
| --- | --- |
| VIRMA sh1 | CCGGCCCAACGATGGCACGAATTACCTCGAGGTAATTCGTGCCATCGTTGGGTTTTTG |
| VIRMA sh2 | CCGGAGGAGTGATCAGTGGATTATTCTCGAGAATAATCCACTGATCACTCCTTTTTTG |
| shCon (targeting *Ath-miR-159*) | CCGGTTTGGATTGAAGGGAGCTCTTCAAGAGAGAGCTCCCTTCAATCCAAACTTTTTTC |
