# Supplementary figures and images for: Overexpression of VIRMA confers vulnerability to breast cancers via the m6A-dependent regulation of unfolded protein response
Source: Cell Mol Life Sci. 2023 May 19;80(6):157. doi: 10.1007/s00018-023-04799-4 (PMC10198946; doi:10.1007/s00018-023-04799-4)

Fig. S1

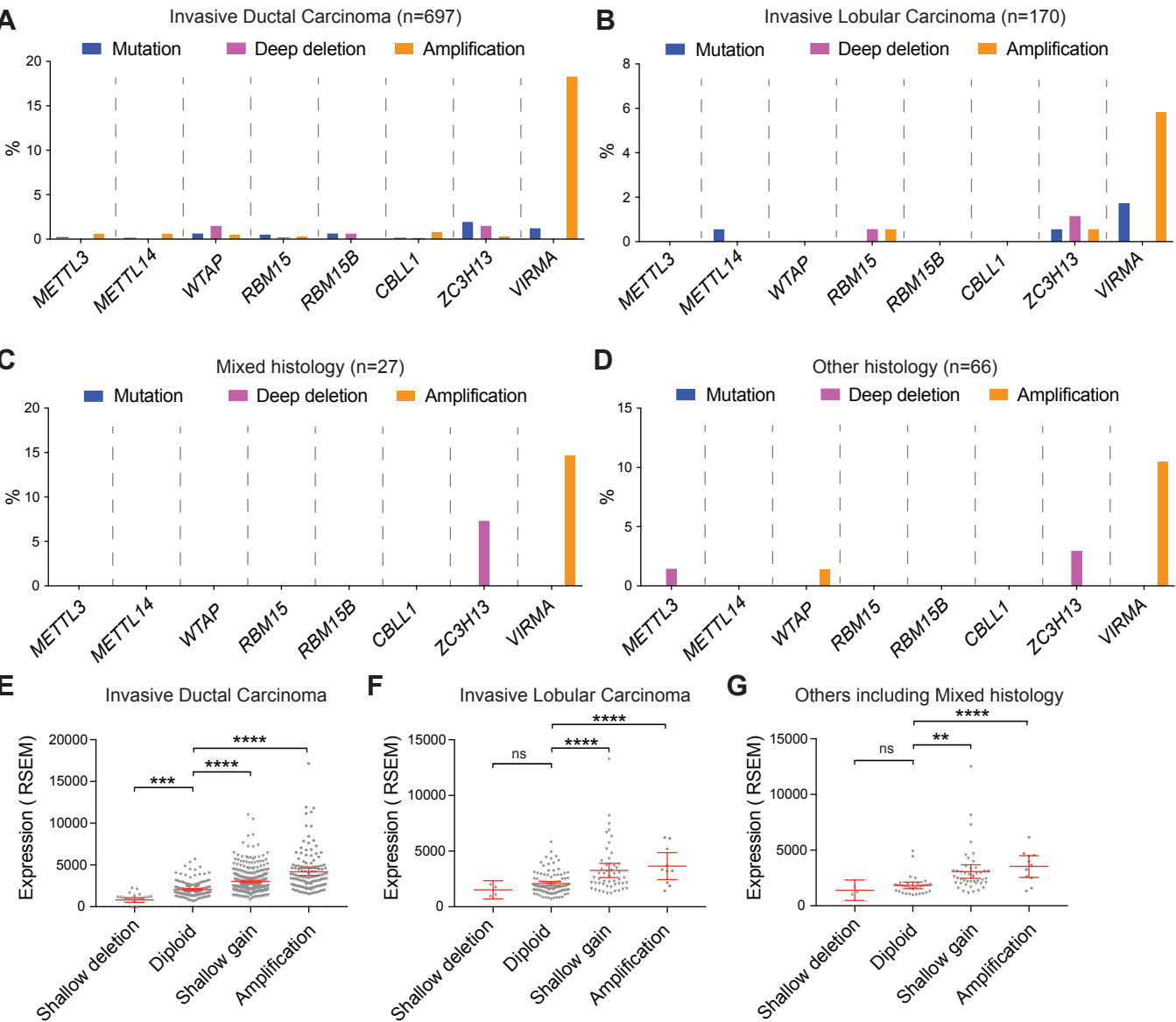

Supplement: Supplementary file 6 — Fig. S1. Alterations of genes encoding components of the m6A writer complex in histologically distinct breast cancers. (A-D) The frequency of mutation and genetic alterations of genes encoding components of the m6A methyltransferase complex in TCGA breast cancers with distinct histological characteristics. (E-G) mRNA expression of VIRMA in breast cancers from the TCGA cohort bearing different genetic alterations of VIRMA stratified based on histological subgroups. All statistical significance shown was determined using one-way ANOVA with Tukey’s test for multiple comparison of the means. **, P <0.01; ***, P <0.001; ****, P <0.0001; ns, not significant. Error bars indicate mean±SEM. (PDF 463 KB) [file 18_2023_4799_MOESM6_ESM.pdf]

**Fig. S2**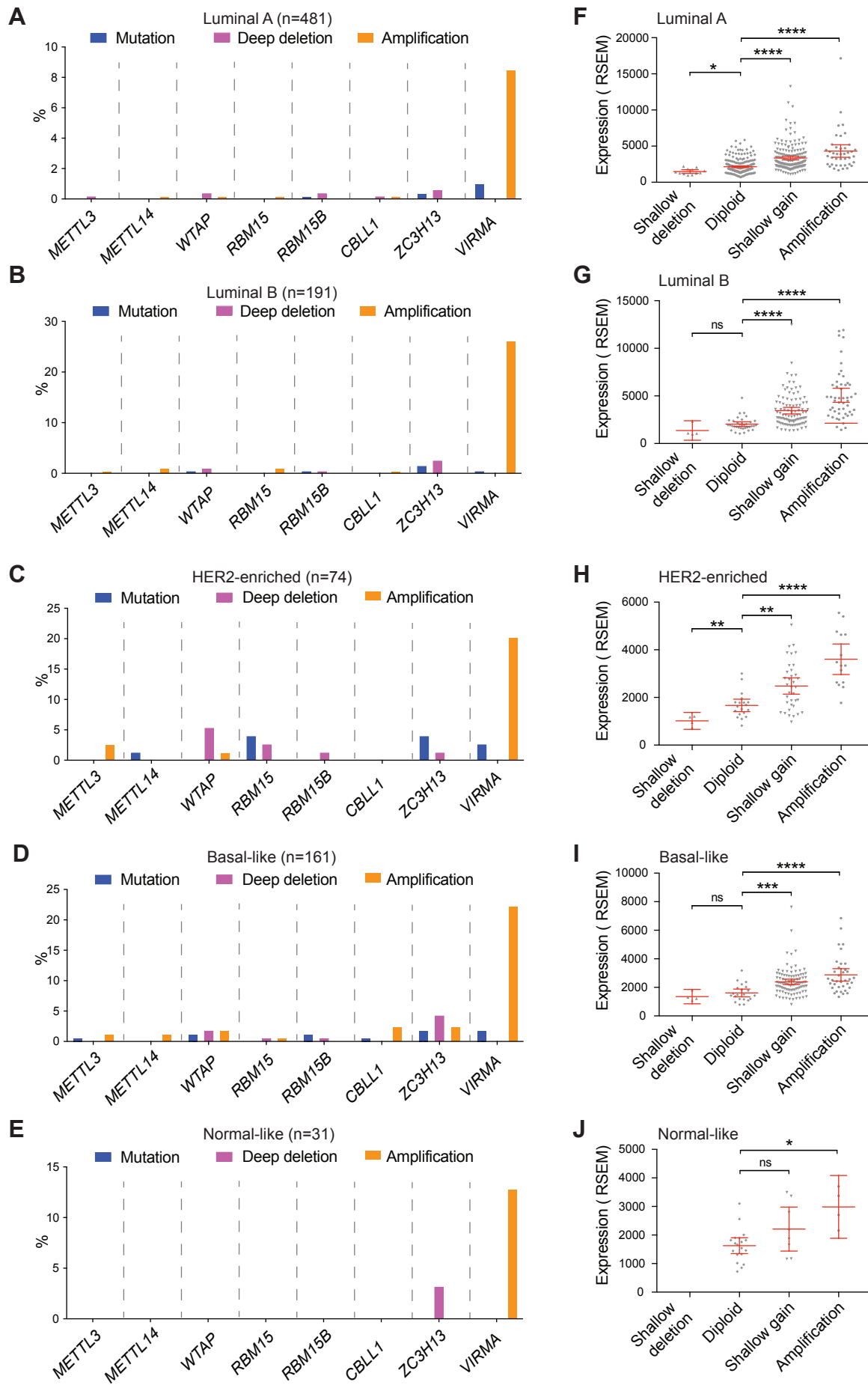

Supplement: Supplementary file 7 — Fig. S2. Alterations of genes encoding components of the m6A writer complex in breast cancers with distinct molecular characteristics. (A–E) The frequency of mutation and genetic alterations of genes encoding components of the m6A methyltransferase complex in TCGA breast cancers with distinct molecular characteristics. (F-J) mRNA expression of VIRMA in TCGA breast cancers with different genetic alterations of VIRMA stratified based on molecular subgroups. All statistical significance shown was determined using one-way ANOVA with Tukey’s test for multiple comparison of the means. *, P <0.05; **, P <0.01; ***, P <0.001; ****, P <0.0001; ns, not significant. Error bars indicate mean±SEM. (PDF 493 KB) [file 18_2023_4799_MOESM7_ESM.pdf]

Fig. S3

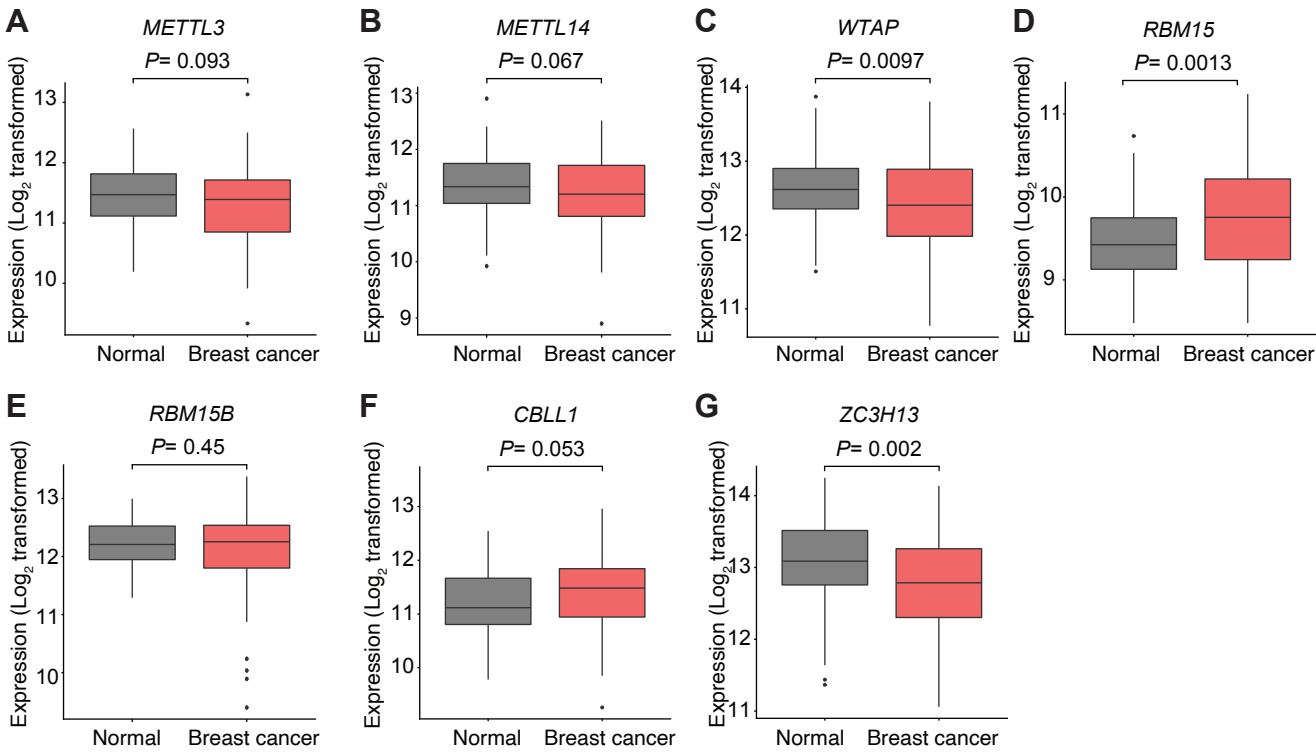

Supplement: Supplementary file 8 — Fig. S3. Expression of genes encoding components of the m6A writer complex in breast cancers and matched normal controls. (A) METTL3. (B) METTL14. (C) WTAP. (D) RBM15. (E) RBM15B. (F) CBLL1. (G) ZC3H13. A total of 79 matched tumours and normal breast tissues were included in this analysis. Statistical significance was determined using unpaired two-tailed Student’s t-test with P <0.05 denoting significance. (PDF 384 KB) [file 18_2023_4799_MOESM8_ESM.pdf]

**Fig. S4**

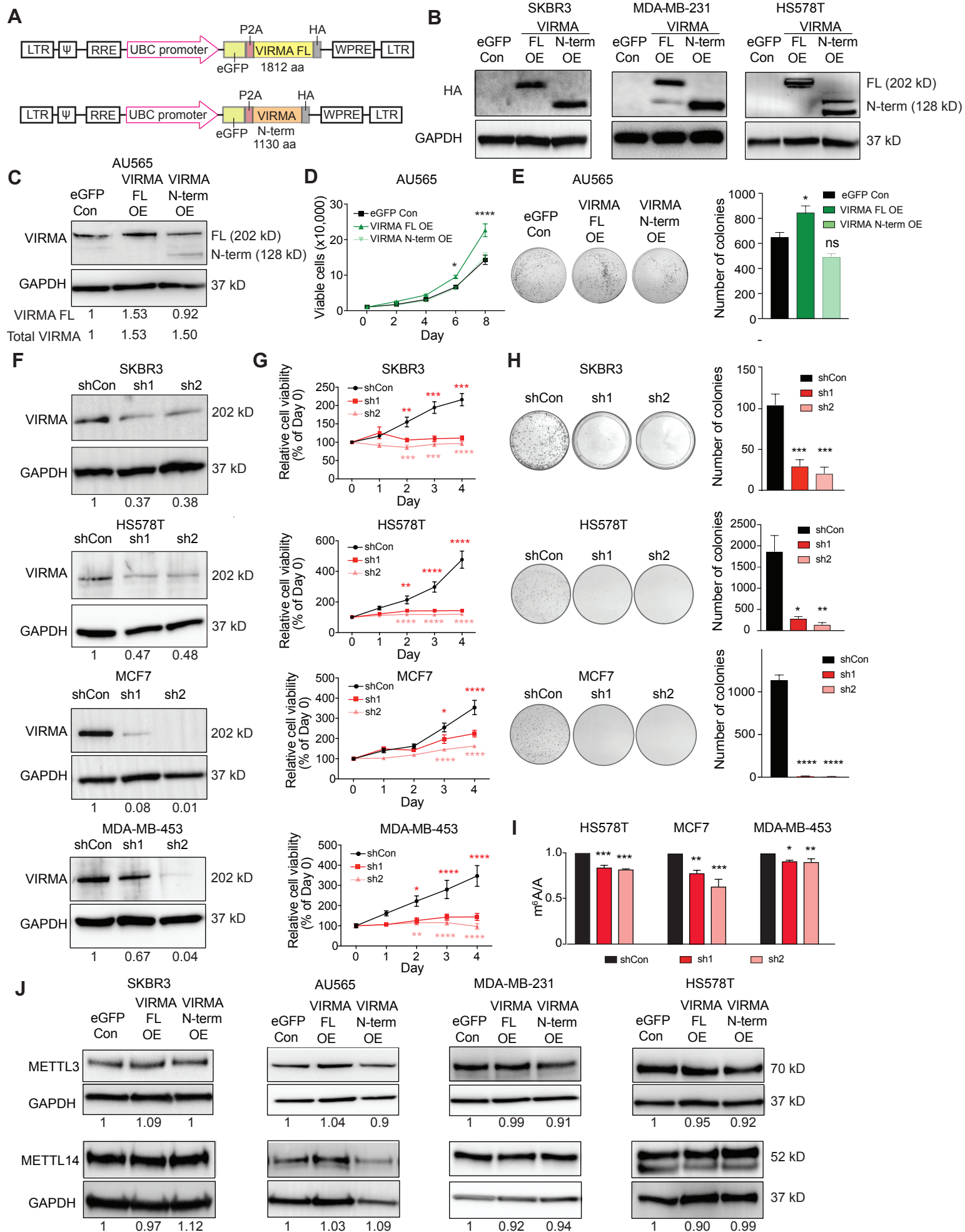

Supplement: Supplementary file 9 — Fig. S4. The effects of VIRMA overexpression and knockdown in breast cancer cell lines. (A) Schematic of lentiviral vector constructs used to induce overexpression of full-length and N-terminal VIRMA in vitro. (B) Western blots showing the detection of HA-tagged full-length (FL OE) and N-terminal VIRMA (N-term OE) in transduced breast cancer cell lines. (C) Representative western blots of full-length (202 kD) and N-terminal VIRMA (128 kD) expression in transduced AU565 breast cancer cell line. FL, full length; N-term, N-terminal. Fold-difference of VIRMA normalised to a loading control (GAPDH) is shown for VIRMA FL OE and N-term OE relative to eGFP control (eGFP Con). (D) Number of viable AU565 breast cancer cells overexpressing full-length VIRMA, N-terminal VIRMA and eGFP alone (eGFP Con) cultured over 8 days. Counting was performed every two days. (E) The colony formation assay performed on AU565 breast cancer cells transduced with lentivirus expressing the full-length or N-terminal VIRMA compared to control cells. Bar plots showing the number of colonies counted for each group (n=3 plates per experimental condition). (F) Representative western blots showing the levels of VIRMA in SKBR3, HS578T, MCF7 and MDA-MB-453 breast cancer cells following lentiviral-mediated transduction with shRNAs against VIRMA (sh1 and sh2) compared to a non-targeting control (shCon). Fold-difference of VIRMA normalised to a loading control (GAPDH) is shown for VIRMA sh1 and sh2 relative to shCon. (G) Cell growth after shRNA-mediated depletion of VIRMA in breast cancer cell lines using the MTT assay. (H) Colony formation in breast cancer cell lines following VIRMA depletion (sh1 and sh2). Bar plots showing the number of colonies counted for each group (n=3 plates per experimental condition). (I) m6A/A ratio on polyadenylated RNAs from HS578T, MCF7 and MDA-MB-453 cells transduced with lentivirus expressing shRNAs (sh1 and sh2) against VIRMA compared to control (shCon). (J) Representative w [file 18_2023_4799_MOESM9_ESM.pdf]

**Fig. S5**

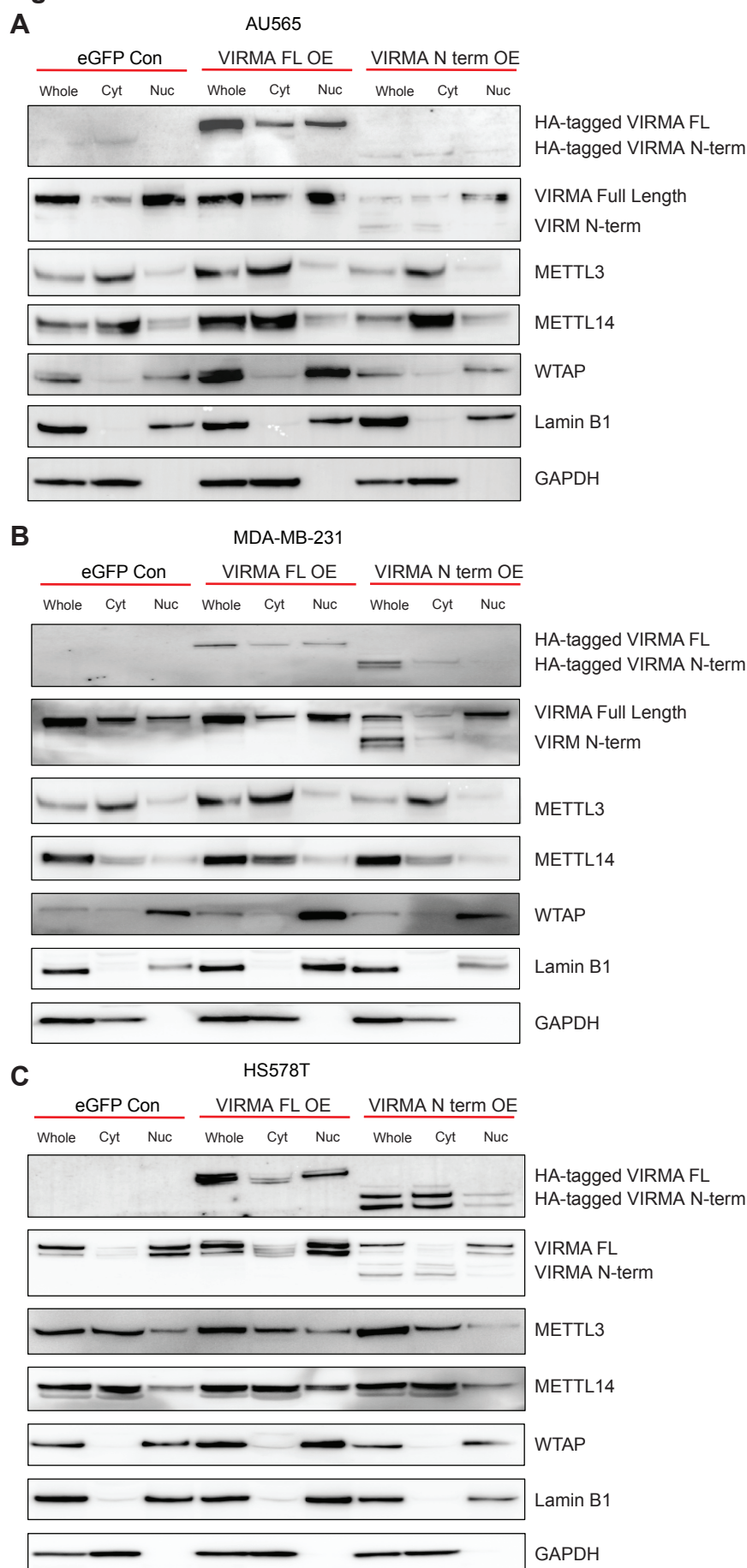

Supplement: Supplementary file 10 — Fig. S5. Distinct localization of full-length and N-terminal VIRMA in cellular compartments. Representative western blot showing the enrichment of VIRMA, METTL3, METTL14 and WTAP in the whole cell (Whole), nuclear (Nuc) and cytoplasmic (Cyt) fractions obtained from (A) AU565, (B) MDA-MB-231 and (C) HS578T cells transduced with lentivirus overexpressing full-length (FL OE) or N-terminal (N-term OE) VIRMA or control (eGFP Con). Lamin B1 and GAPDH were included to confirm the purity of nuclear and cytoplasmic extract respectively. (PDF 2116 KB) [file 18_2023_4799_MOESM10_ESM.pdf]

**Fig. S6**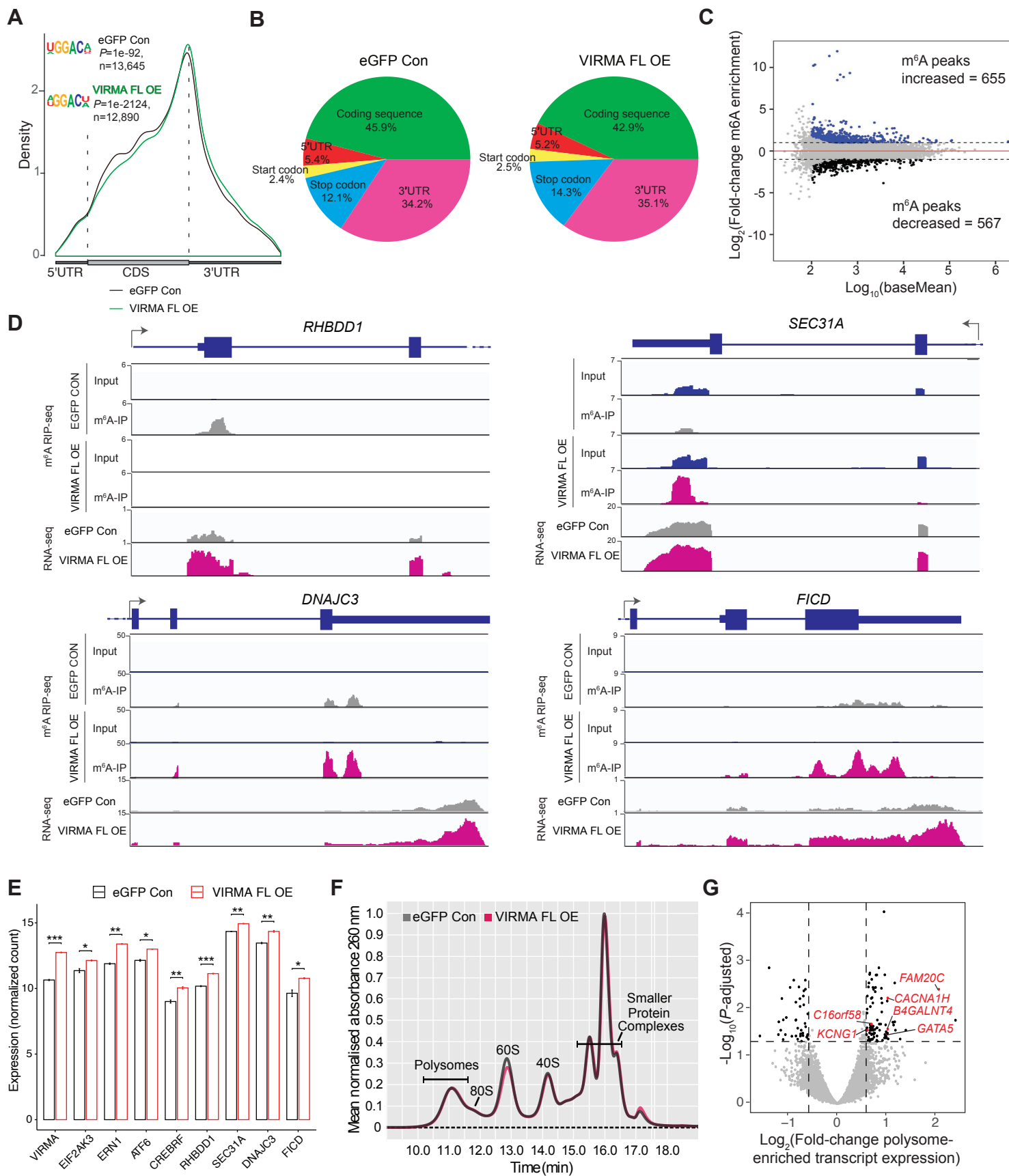

Supplement: Supplementary file 11 — Fig. S6. The association between full-length VIRMA overexpression and unfolded protein response. (A) Metagene plots showing locus- and motif-specific enrichment of m6A peaks detected using m6A-RIP-sequencing in SKBR3 cells overexpressing full-length VIRMA (FL OE) and control cells (eGFP Con). (B) Distribution of m6A peaks at different genomic loci in SKBR3 cells overexpressing full-length VIRMA and control cells. (C) Distribution of increased and decreased m6A peaks following overexpression of full-length VIRMA in SKBR3 cells. Significantly increased or decreased peaks are represented in blue and black, respectively (log2 fold-change m6A enrichment > or < 2, P adjusted by Benjamini–Hochberg correction <0.05). (D) Integrative genome viewer plots showing examples of genes encoding unfolded protein response regulators with differential enrichment of m6A and mRNA expression following overexpression of full-length VIRMA (FL OE) in SKBR3 cells. (E) mRNA expression of UPR-related genes in VIRMA FL OE and control (eGFP Con) measured by mRNA-seq. Statistical significance was determined using unpaired two-tailed Student’s t-test. Data are from 3 biological replicates and show mean±SEM. *, P <0.05; **, P <0.01; ***, P <0.001; ****, P <0.0001. (F) Ribosome profile of SKBR3 cells transduced with lentivirus overexpressing full-length VIRMA and eGFP alone (eGFP Con). (G) Volcano plots showing differentially enriched genes in the polysome fractions following the overexpression of full-length VIRMA in SKBR3 cells compared to control. Genes with m6A methylation are highlighted in red. (PDF 2305 KB) [file 18_2023_4799_MOESM11_ESM.pdf]

**Fig. S7**

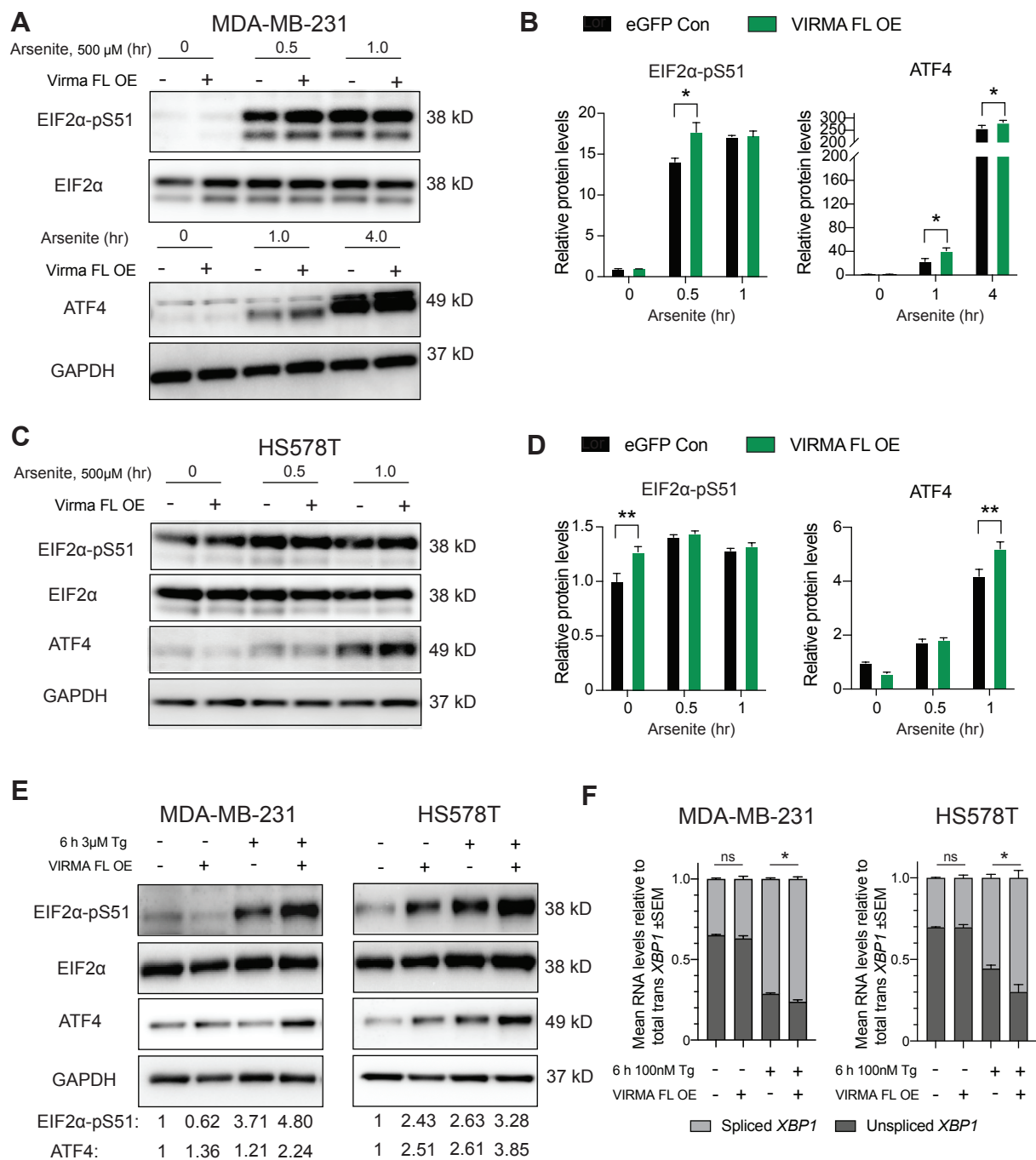

Supplement: Supplementary file 12 — Fig. S7. Enhancement of UPR signaling under stress in MDA-MB-231 and HS578T cells consequent to full-length VIRMA overexpression. (A) Representative western blots showing the levels of UPR proteins in MDA-MB-231 cells transduced with lentivirus expressing full-length VIRMA at baseline and after exposure to sodium arsenite. (B) Relative levels of EIF2α-pS51 and ATF4 proteins in full-length VIRMA overexpressing MDA-MB231 and control cells exposed to sodium arsenite. Representative western blots as in (A) performed for HS578T cells. (D) Relative levels of EIF2α-pS51 and ATF4 proteins in full-length VIRMA overexpressing HS578T and control cells exposed to sodium arsenite. (E) Western blots showing the levels of UPR proteins in VIRMA-overexpressing MDA-MB-231 and HS578T cells at baseline and after stimulation with 3 μM Thapsigargin (Tg) for 6 hours. Fold-change of these proteins normalised to respective loading control (EIF2α or GAPDH) is shown. (F) The proportion of spliced versus unspliced XBP1 transcripts in full-length VIRMA-overexpressing MDA-MB-231 and HS578T cells at baseline and after stimulation with Thapsigargin (Tg). For bar plots, data are from 3 biological replicates and show mean±SEM. All statistical significance shown was determined using two-way ANOVA with multiple comparisons of the means performed using the Tukey’s test. *, P <0.05; **, P <0.01; ns, not significant (PDF 1245 KB) [file 18_2023_4799_MOESM12_ESM.pdf]
