# Supplementary material for: Overexpression of VIRMA confers vulnerability to breast cancers via the m6A-dependent regulation of unfolded protein response
Source: Cell Mol Life Sci. 2023 May 19;80(6):157. doi: 10.1007/s00018-023-04799-4 (PMC10198946; doi:10.1007/s00018-023-04799-4)
Supplement: Supplementary file 13 — Supplementary Methods (DOCX 17 KB) [file 18_2023_4799_MOESM13_ESM.docx]

**Construction of VIRMA FL- and VIRMA N-term-overexpressing vectors**

The following DNA sequences were synthesized to incorporate VIRMA FL or N-term, eGFP separated by a p2A sequence, HA-tag, flexible linker and additional sequences to generate compatible ends to clone into a FUW-myc empty vector that was kindly provided by Dr Victor Anggono (University of Queensland). These insert sequences were digested with SpeI-HF and NotI to yield compatible ends to clone into FUW-myc digested with XbaI and NotI. All restriction enzymes are available from New England Biolabs.

**VIRMA FL:**

Compatible ends, eGPF-p2A, VIRMA FL, flexible linker, HA-tag with stop codon

tctttctactagtggatccccgaaccgacagtcggtctcttcaccaaggccattcgcgccaccATGGTGAGCAAGGGCGAGGAGCTGTTCACCGGGGTGGTGCCCATCCTGGTCGAGCTGGACGGCGACGTAAACGGCCACAAGTTCAGCGTGTCCGGCGAGGGCGAGGGCGATGCCACCTACGGCAAGCTGACCCTGAAGTTCATCTGCACCACCGGCAAGCTGCCCGTGCCCTGGCCCACCCTCGTGACCACCCTGACCTACGGCGTGCAGTGCTTCAGCCGCTACCCCGACCACATGAAGCAGCACGACTTCTTCAAGTCCGCCATGCCCGAAGGCTACGTCCAGGAGCGCACCATCTTCTTCAAGGACGACGGCAACTACAAGACCCGCGCCGAGGTGAAGTTCGAGGGCGACACCCTGGTGAACCGCATCGAGCTGAAGGGCATCGACTTCAAGGAGGACGGCAACATCCTGGGGCACAAGCTGGAGTACAACTACAACAGCCACAACGTCTATATCATGGCCGACAAGCAGAAGAACGGCATCAAGGTGAACTTCAAGATCCGCCACAACATCGAGGACGGCAGCGTGCAGCTCGCCGACCACTACCAGCAGAACACCCCCATCGGCGACGGCCCCGTGCTGCTGCCCGACAACCACTACCTGAGCACCCAGTCCGCCCTGAGCAAAGACCCCAACGAGAAGCGCGATCACATGGTCCTGCTGGAGTTCGTGACCGCCGCCGGGATCACTCTCGGCATGGACGAGCTGTACAAGTCAGGATCCGGAGCCACGAACTTCTCTCTGTTAAAGCAAGCAGGAGACGTGGAAGAAAACCCCGGGCCCATGGCGGTGGACTCGGCGATGGAGCTGTTATTTTTAGATACTTTTAAACACCCGAGCGCTGAGCAAAGTTCTCATATAGATGTGGTTCGTTTTCCATGTGTGGTTTATATCAATGAAGTCCGAGTCATACCCCCAGGAGTAAGAGCCCATAGCAGTCTGCCAGACAATAGAGCATATGGAGAGACATCTCCCCATACATTTCAATTAGACTTATTCTTCAACAATGTAAGCAAACCAAGTGCCCCTGTTTTCGATAGGTTGGGAAGCCTGGAATATGATGAGAATACTTCCATCATCTTTAGACCTAACTCAAAGGTGAATACTGATGGTCTGGTGCTAAGAGGCTGGTATAACTGTCTGACACTGGCAATATATGGATCAGTGGATAGAGTGATAAGTCATGACAGAGACTCTCCACCACCACCACCTCCACCGCCACCACCTCCCCAGCCACAACCAAGTTTGAAAAGGAATCCAAAACATGCTGATGGGGAGAAAGAAGATCAGTTTAATGGAAGCCCTCCAAGACCACAGCCAAGGGGACCAAGAACTCCTCCAGGACCCCCTCCACCTGATGATGATGAAGATGATCCTGTGCCTCTGCCAGTGTCTGGTGACAAGGAAGAGGAT

GCTCCTCATAGAGAAGATTACTTTGAGCCCATTTCTCCTGATCGGAATTCTGTTCCCCAGGAAGGGCAATATTCTGATGAAGGAGAAGTAGAAGAGGAACAACAAGAAGAAGGAGAAGAAGATGAAGATGATGTGGATGTAGAGGAAGAAGAGGATGAGGATGAGGATGATCGACGAACAGTAGACAGTATTCCTGAGGAGGAAGAGGAAGATGAAGAGGAAGAAGGTGAAGAGGATGAAGAAGGTGAAGGGGATGATGGTTATGAACAAATTTCCAGTGATGAAGATGGAATTGCTGACTTGGAACGTGAAACATTTAAGTATCCAAACTTTGATGTTGAATACACTGCTGAAGACTTAGCTTCAGTTCCTCCTATGACATATGATCCATATGACAGGGAGCTTGTACCACTCTTATACTTCAGTTGTCCATACAAGACTACTTTTGAAATTGAAATCAGTAGAATGAAGGATCAAGGTCCAGATAAAGAAAATTCAGGGGCAATCGAAGCCTCAGTGAAGTTAACAGAACTCTTAGATTTGTATAGAGAAGATAGAGGTGCAAAATGGGTAACAGCTTTAGAAGAAATTCCAAGTTTAATAATAAAAGGGTTAAGCTATTTGCAATTGAAAAACACAAAACAAGACTCCCTTGGCCAGTTGGTAGACTGGACCATGCAAGCTTTAAATTTACAAGTAGCGCTTCGCCAACCTATCGCCTTAAATGTTCGACAGCTCAAAGCTGGGACCAAATTAGTGTCCTCACTAGCAGAATGTGGGGCTCAAGGAGTTACAGGACTGCTACAAGCAGGAGTGATCAGTGGATTATTTGAACTTCTGTTTGCTGATCACGTATCATCTTCTCTTAAGTTAAATGCTTTTAAAGCTTTGGACAGTGTCATTAGTATGACAGAAGGAATGGAAGCTTTTTTAAGAGGTAGGCAGAATGAAAAAAGTGGTTATCAAAAGCTTCTGGAACTCATACTTTTAGATCAGACTGTGAGGGTTGTTACTGCTGGTTCAGCTATTCTCCAAAAATGCCATTTCTATGAAGTCTTGTCAGAGATTAAAAGACTTGGTGACCATTTAGCAGAGAAGACTTCATCTCTTCCTAACCACAGTGAACCTGATCACGACACAGATGCTGGACTTGAGAGAACAAACCCAGAATATGAAAATGAGGTGGAAGCTTCTATGGATATGGATCTTTTGGAATCCTCAAATATAAGTGAAGGGGAAATAGAAAGGCTTATTAACCTCCTAGAAGAAGTTTTTCATTTAATGGAAACTGCCCCTCATACAATGATCCAACAACCTGTTAAGTCTTTCCCAACGATGGCACGAATTACTGGACCTCCAGAGAGGGATGATCCATACCCTGTTCTCTTTAGATATCTTCACAGTCATCACTTCTTGGAGTTGGTTACCTTGCTTCTGTCAATTCCAGTAACAAGTGCTCACCCTGGTGTGCTGCAAGCCACAAAAGATGTTTTGAAGTTTCTTGCACAGTCACAGAAGGGTCTTCTTTTTTTTATGTCGG

AATATGAAGCAACAAATTTATTGATCCGAGCTCTGTGTCACTTTTATGATCAAGATGAGGAGGAAGGTCTCCAATCTGATGGTGTTATTGATGATGCATTTGCCTTGTGGCTACAGGACTCAACACAGACATTGCAATGTATTACAGAACTGTTCAGCCATTTTCAGCGTTGTACAGCCAGTGAAGAAACAGACCATTCAGATCTCTTGGGAACCCTGCACAATCTTTATTTGATTACTTTTAATCCTGTGGGAAGATCAGCTGTTGGCCATGTTTTTAGTCTGGAGAAAAATCTCCAAAGTCTTATTACTCTAATGGAGTACTATTCCAAAGAAGCCTTGGGTGATTCCAAATCTAAGAAGTCAGTAGCTTATAATTACGCATGCATACTTATTTTGGTGGTGGTTCAGTCTTCCAGTGATGTTCAAATGCTAGAACAACATGCAGCATCTCTCTTGAAGCTTTGTAAAGCAGATGAAAATAATGCTAAATTGCAAGAACTTGGCAAGTGGCTTGAACCTCTGAAAAACCTTAGATTTGAAATTAACTGCATCCCAAACTTAATTGAGTATGTTAAGCAGAATATCGATAACTTGATGACCCCAGAAGGAGTTGGCCTTACCACTGCCTTACGTGTTCTCTGTAATGTTGCATGCCCACCACCTCCTGTTGAAGGTCAACAGAAAGATCTGAAATGGAATCTTGCCGTTATTCAGCTTTTTTCTGCTGAAGGAATGGACACGTTTATTCGAGTTCTGCAAAAATTGAACAGTATTCTGACTCAGCCTTGGAGGCTCCATGTCAACATGGGGACTACCCTTCACAGAGTTACTACTATTTCAATGGCTCGCTGCACACTCACTCTTCTTAAAACTATGTTAACGGAACTCCTGAGAGGTGGATCCTTTGAGTTTAAGGACATGCGTGTTCCTTCAGCGCTTGTTACTTTACATATGCTCCTGTGCTCTATCCCCCTCTCAGGTCGTTTGGATAGTGATGAACAGAAAATTCAGAATGATATCATTGATATTTTACTGACTTTTACACAAGGAGTTAATGAAAAACTCACAATCTCAGAAGAGACTCTGGCCAATAATACTTGGTCTTTAATGTTAAAAGAAGTTCTTTCTTCAATCTTGAAGGTTCCTGAAGGATTTTTTTCTGGACTCATACTCCTTTCAGAGCTGCTGCCTCTTCCATTGCCCATGCAAACAACTCAGGTTATTGAGCCACATGATATATCAGTGGCACTCAACACCCGAAAATTGTGGAGCATGCACCTTCATGTTCAAGCAAAGTTGCTCCAAGAAATAGTTCGCTCTTTCTCTGGCACAACCTGCCAGCCCATTCAACATATGTTACGGCGTATTTGTGTTCAATTGTGTGACCTTGCCTCACCAACTGCACTTCTGATTATGAGAACTGTGTTGGATTTGATTGTAGAAGACTTGCAAAGCACTTCAGAAGATAAAGAAAAACAGTATACTAGCCAAACCACCAGGTTGCTTGCTCTTCTTGATGCTCTGGCTTCACACAAAGCTTGTAAATTAGCTATTTTGCATCTAATTAATGGAACTATTAAAGGTGATGAAAGATATGCAGAGATATTCCAGGATCTTTTAGCTTTGGTGCGGTCTCCTGGAGACAGTGTTATTCGCCAACAGTGTGTTGAATATGTCACATCCATTTTGCAGTCTCTCTGTGATCAGGACATTGCACTTATCTTACCAAGCTCTTCTGAAGGTTCTATTTCTGAACTGGAGCAGCTCTCCAATTCTCTACCAAATAAAGAATTGATGACCTCAATCTGTGACTGTCTGTTGGCTACGCTAGCTAACTCTGAGAGCAGTTACAACTGTTTACTGACATGTGTCAGAACAATGATGTTTCTTGCAGAGCATGATTATGGATTATTTCATTTAAAAAGTTCTTTAAGGAAAAACAGTAGTGCTCTGCATAGTTTACTGAAACGAGTGGTCAGCACATTTAGTAAGGACACAGGAGAGCTTGCATCTTCATTTTTAGAATTTATGAGACAAATTCTTAACTCTGACACAATTGGATGCTGTGGAGATGATAATGGTCTCATGGAAGTAGAGGGAGCTCATACATCACGGACGATGAGTATTAATGCTGCAGAGTTAAAACAGCTTCTACAAAGCAAAGAAGAAAGTCCAGAAAATTTGTTCCTTGAACTAGAGAAGCTTGTTTTGGAACATTCAAAAGATGATGACAATCTGGATTCTTTGTTGGACAGTGTAGTTGGACTTAAGCAGATGCTGGAGTCATCAGGTGACCCTTTACCTCTCAGTGACCAGGATGTAGAACCAGTACTTTCAGCTCCAGAATCTCTTCAGAATCTGTTTAACAATAGGACTGCCTATGTGCTTGCTGATGTCATGGATGATCAGTTGAAATCTATGTGGTTCACTCCATTTCAGGCTGAAGAGATAGATACAGATCTGGATTTGGTAAAGGTTGACTTAATTGAACTCTCTGAAAAATGCTGTAGTGACTTTGATTTGCACTCAGAATTAGAGCGCTCATTTTTGTCAGAACCATCATCTCCAGGAAGAACCAAGACTACTAAAGGATTCAAACTTGGGAAGCACAAGCATGAGACCTTTATAACGTCAAGTGGAAAATCTGAATACATTGAACCTGCCAAAAGAGCTCATGTTGTGCCACCACCAAGAGGAAGGGGCAGGGGAGGATTTGGACAGGGTATACGACCTCATGATATTTTTCGTCAGAGAAAACAGAACACAAGTAGACCACCATCTATGCATGTGGATGACTTTGTTGCTGCTGAAAGTAAAGAAGTGGTTCCTCAAGATGGAATACCTCCACCAAAACGGCCACTCAAAGTATCACAGAAGATTTCTTCCCGTGGTGGGTTTTCAGGCAATAGAGGAGGACGGGGTGCTTTCCACAGTCAGAATAGGTTTTTCACACCACCTGCTTCAAAAGGAAACTACAGTCGTCGGGAAGGAACAAGAGGCTCCAGTTGGAGTGCTCAGAATACTCCTCGAGGAAATTACAATGAAAGTCGTGGAGGCCAGAGCAATTTTAACAGAGGCCCTCTTCCACCATTACGACCCCTTAGTTCTACAGGTTACCGCCCAAGTCCTCGGGACCGTGCTTCTAGAGGTCGTGGGGGACTTGGACCTTCCTGGGCTAGTGCAAATAGCGGCAGTGGAGGCTCAAGAGGAAAGTTTGTTAGTGGAGGCAGTGGTAGAGGTCGTCATGTACGCTCCTTTACACGATCGGGCTCAAGCTCTGGCTCGAGTCTAGACGCTTCTTGCTATCCTTATGACGTGCCTGACTATGCCAGCCTGTGAgtttaaacgcggccgctatttgt

**N-term VIRMA:**

Compatible ends, eGPF-p2A, VIRMA N-term, flexible linker, HA-tag with stop codon

tctttctactagtggatccccgaaccgacagtcggtctcttcaccaaggccattcgcgccaccATGGTGAGCAAGGGCGAGGAGCTGTTCACCGGGGTGGTGCCCATCCTGGTCGAGCTGGACGGCGACGTAAACGGCCACAAGTTCAGCGTGTCCGGCGAGGGCGAGGGCGATGCCACCTACGGCAAGCTGACCCTGAAGTTCATCTGCACCACCGGCAAGCTGCCCGTGCCCTGGCCCACCCTCGTGACCACCCTGACCTACGGCGTGCAGTGCTTCAGCCGCTACCCCGACCACATGAAGCAGCACGACTTCTTCAAGTCCGCCATGCCCGAAGGCTACGTCCAGGAGCGCACCATCTTCTTCAAGGACGACGGCAACTACAAGACCCGCGCCGAGGTGAAGTTCGAGGGCGACACCCTGGTGAACCGCATCGAGCTGAAGGGCATCGACTTCAAGGAGGACGGCAACATCCTGGGGCACAAGCTGGAGTACAACTACAACAGCCACAACGTCTATATCATGGCCGACAAGCAGAAGAACGGCATCAAGGTGAACTTCAAGATCCGCCACAACATCGAGGACGGCAGCGTGCAGCTCGCCGACCACTACCAGCAGAACACCCCCATCGGCGACGGCCCCGTGCTGCTGCCCGACAACCACTACCTGAGCACCCAGTCCGCCCTGAGCAAAGACCCCAACGAGAAGCGCGATCACATGGTCCTGCTGGAGTTCGTGACCGCCGCCGGGATCACTCTCGGCATGGACGAGCTGTACAAGTCAGGATCCGGAGCCACGAACTTCTCTCTGTTAAAGCAAGCAGGAGACGTGGAAGAAAACCCCGGGCCCATGGCGGTGGACTCGGCGATGGAGCTGTTATTTTTAGATACTTTTAAACACCCGAGCGCTGAGCAAAGTTCTCATATAGATGTGGTTCGTTTTCCATGTGTGGTTTATATCAATGAAGTCCGAGTCATACCCCCAGGAGTAAGAGCCCATAGCAGTCTGCCAGACAATAGAGCATATGGAGAGACATCTCCCCATACATTTCAATTAGACTTATTCTTCAACAATGTAAGCAAACCAAGTGCCCCTGTTTTCGATAGGTTGGGAAGCCTGGAATATGATGAGAATACTTCCATCATCTTTAGACCTAACTCAAAGGTGAATACTGATGGTCTGGTGCTAAGAGGCTGGTATAACTGTCTGACACTGGCAATATATGGATCAGTGGATAGAGTGATAAGTCATGACAGAGACTCTCCACCACCACCACCTCCACCGCCACCACCTCCCCAGCCACAACCAAGTTTGAAAAGGAATCCAAAACATGCTGATGGGGAGAAAGAAGATCAGTTTAATGGAAGCCCTCCAAGACCACAGCCAAGGGGACCAAGAACTCCTCCAGGACCCCCTCCACCTGATGATGATGAAGATGATCCTGTGCCTCTGCCAGTGTCTGGTGACAAGGAAGAGGAT

GCTCCTCATAGAGAAGATTACTTTGAGCCCATTTCTCCTGATCGGAATTCTGTTCCCCAGGAAGGGCAATATTCTGATGAAGGAGAAGTAGAAGAGGAACAACAAGAAGAAGGAGAAGAAGATGAAGATGATGTGGATGTAGAGGAAGAAGAGGATGAGGATGAGGATGATCGACGAACAGTAGACAGTATTCCTGAGGAGGAAGAGGAAGATGAAGAGGAAGAAGGTGAAGAGGATGAAGAAGGTGAAGGGGATGATGGTTATGAACAAATTTCCAGTGATGAAGATGGAATTGCTGACTTGGAACGTGAAACATTTAAGTATCCAAACTTTGATGTTGAATACACTGCTGAAGACTTAGCTTCAGTTCCTCCTATGACATATGATCCATATGACAGGGAGCTTGTACCACTCTTATACTTCAGTTGTCCATACAAGACTACTTTTGAAATTGAAATCAGTAGAATGAAGGATCAAGGTCCAGATAAAGAAAATTCAGGGGCAATCGAAGCCTCAGTGAAGTTAACAGAACTCTTAGATTTGTATAGAGAAGATAGAGGTGCAAAATGGGTAACAGCTTTAGAAGAAATTCCAAGTTTAATAATAAAAGGGTTAAGCTATTTGCAATTGAAAAACACAAAACAAGACTCCCTTGGCCAGTTGGTAGACTGGACCATGCAAGCTTTAAATTTACAAGTAGCGCTTCGCCAACCTATCGCCTTAAATGTTCGACAGCTCAAAGCTGGGACCAAATTAGTGTCCTCACTAGCAGAATGTGGGGCTCAAGGAGTTACAGGACTGCTACAAGCAGGAGTGATCAGTGGATTATTTGAACTTCTGTTTGCTGATCACGTATCATCTTCTCTTAAGTTAAATGCTTTTAAAGCTTTGGACAGTGTCATTAGTATGACAGAAGGAATGGAAGCTTTTTTAAGAGGTAGGCAGAATGAAAAAAGTGGTTATCAAAAGCTTCTGGAACTCATACTTTTAGATCAGACTGTGAGGGTTGTTACTGCTGGTTCAGCTATTCTCCAAAAATGCCATTTCTATGAAGTCTTGTCAGAGATTAAAAGACTTGGTGACCATTTAGCAGAGAAGACTTCATCTCTTCCTAACCACAGTGAACCTGATCACGACACAGATGCTGGACTTGAGAGAACAAACCCAGAATATGAAAATGAGGTGGAAGCTTCTATGGATATGGATCTTTTGGAATCCTCAAATATAAGTGAAGGGGAAATAGAAAGGCTTATTAACCTCCTAGAAGAAGTTTTTCATTTAATGGAAACTGCCCCTCATACAATGATCCAACAACCTGTTAAGTCTTTCCCAACGATGGCACGAATTACTGGACCTCCAGAGAGGGATGATCCATACCCTGTTCTCTTTAGATATCTTCACAGTCATCACTTCTTGGAGTTGGTTACCTTGCTTCTGTCAATTCCAGTAACAAGTGCTCACCCTGGTGTGCTGCAAGCCACAAAAGATGTTTTGAAGTTTCTTGCACAGTCACAGAAGGGTCTTCTTTTTTTTATGTCGG

AATATGAAGCAACAAATTTATTGATCCGAGCTCTGTGTCACTTTTATGATCAAGATGAGGAGGAAGGTCTCCAATCTGATGGTGTTATTGATGATGCATTTGCCTTGTGGCTACAGGACTCAACACAGACATTGCAATGTATTACAGAACTGTTCAGCCATTTTCAGCGTTGTACAGCCAGTGAAGAAACAGACCATTCAGATCTCTTGGGAACCCTGCACAATCTTTATTTGATTACTTTTAATCCTGTGGGAAGATCAGCTGTTGGCCATGTTTTTAGTCTGGAGAAAAATCTCCAAAGTCTTATTACTCTAATGGAGTACTATTCCAAAGAAGCCTTGGGTGATTCCAAATCTAAGAAGTCAGTAGCTTATAATTACGCATGCATACTTATTTTGGTGGTGGTTCAGTCTTCCAGTGATGTTCAAATGCTAGAACAACATGCAGCATCTCTCTTGAAGCTTTGTAAAGCAGATGAAAATAATGCTAAATTGCAAGAACTTGGCAAGTGGCTTGAACCTCTGAAAAACCTTAGATTTGAAATTAACTGCATCCCAAACTTAATTGAGTATGTTAAGCAGAATATCGATAACTTGATGACCCCAGAAGGAGTTGGCCTTACCACTGCCTTACGTGTTCTCTGTAATGTTGCATGCCCACCACCTCCTGTTGAAGGTCAACAGAAAGATCTGAAATGGAATCTTGCCGTTATTCAGCTTTTTTCTGCTGAAGGAATGGACACGTTTATTCGAGTTCTGCAAAAATTGAACAGTATTCTGACTCAGCCTTGGAGGCTCCATGTCAACATGGGGACTACCCTTCACAGAGTTACTACTATTTCAATGGCTCGCTGCACACTCACTCTTCTTAAAACTATGTTAACGGAACTCCTGAGAGGTGGATCCTTTGAGTTTAAGGACATGCGTGTTCCTTCAGCGCTTGTTACTTTACATATGCTCCTGTGCTCTATCCCCCTCTCAGGTCGTTTGGATAGTGATGAACAGAAAATTCAGAATGATATCATTGATATTTTACTGACTTTTACACAAGGAGTTAATGAAAAACTCACAATCTCAGAAGAGACTCTGGCCAATAATACTTGGTCTTTAATGTTAAAAGAAGTTCTTTCTTCAATCTTGAAGGTTCCTGAAGGATTTTTTTCTGGACTCATACTCCTTTCAGAGCTGCTGCCTCTTCCATTGCCCATGCAAACAACTCAGGTATCACTTCCATATAACATGCATCTTATAAATGACTGCAGTAACACTTTTTCGGGCTCAAGCTCTGGCTCGAGTCTAGACGCTTCTTGCTATCCTTATGACGTGCCTGACTATGCCAGCCTGTGAgtttaaacgcggccgctatttgt
